# The SMC5/6 complex compacts and silences unintegrated HIV-1 DNA and is antagonized by Vpr

**DOI:** 10.1016/j.chom.2021.03.001

**Published:** 2021-05-12

**Authors:** Liane Dupont, Stuart Bloor, James C. Williamson, Sergio Martínez Cuesta, Raven Shah, Ana Teixeira-Silva, Adi Naamati, Edward J.D. Greenwood, Stefan G. Sarafianos, Nicholas J. Matheson, Paul J. Lehner

**Affiliations:** 1 Cambridge Institute for Therapeutic Immunology & Infectious Disease, Jeffrey Cheah Biomedical Centre, Cambridge Biomedical Campus, University of Cambridge, Cambridge CB2 0AW, UK; 2 Cancer Research UK Cambridge Institute, Li Ka Shing Centre, Cambridge CB2 0RE, UK; 3 Laboratory of Biochemical Pharmacology, Department of Pediatrics, Emory University School of Medicine and Children’s Healthcare of Atlanta, Atlanta, GA 30322, USA

**Keywords:** HIV-1, SMC5-SMC6 complex, SLF2, Vpr, viral gene silencing, unintegrated lentivirus, Extrachromosomal DNA, chromatin compaction, CRISPR-Cas9 knockout screen, ATAC-seq

## Abstract

Silencing of nuclear DNA is an essential feature of innate immune responses to invading pathogens. Early in infection, unintegrated lentiviral cDNA accumulates in the nucleus yet remains poorly expressed. In HIV-1-like lentiviruses, the Vpr accessory protein enhances unintegrated viral DNA expression, suggesting Vpr antagonizes cellular restriction. We previously showed how Vpr remodels the host proteome, identifying multiple cellular targets. We now screen these using a targeted CRISPR-Cas9 library and identify SMC5-SMC6 complex localization factor 2 (SLF2) as the Vpr target responsible for silencing unintegrated HIV-1. SLF2 recruits the SMC5/6 complex to unintegrated lentiviruses, and depletion of SLF2, or the SMC5/6 complex, increases viral expression. ATAC-seq demonstrates that Vpr-mediated SLF2 depletion increases chromatin accessibility of unintegrated virus, suggesting that the SMC5/6 complex compacts viral chromatin to silence gene expression. This work implicates the SMC5/6 complex in nuclear immunosurveillance of extrachromosomal DNA and defines its targeting by Vpr as an evolutionarily conserved antagonism.

## Highlights

• Gene expression from unintegrated HIV-1 DNA species is restricted by the host cell
• Vpr antagonizes silencing by degrading cellular targets via the CRL4^DCAF1^ E3 ligase
• A custom “Vpr library” CRISPR-Cas9 knockout screen implicates SLF2 in silencing
• SLF2 recruits the SMC5/6 complex to compact and silence unintegrated HIV-1 DNA

## Graphical abstract

**Figure:**
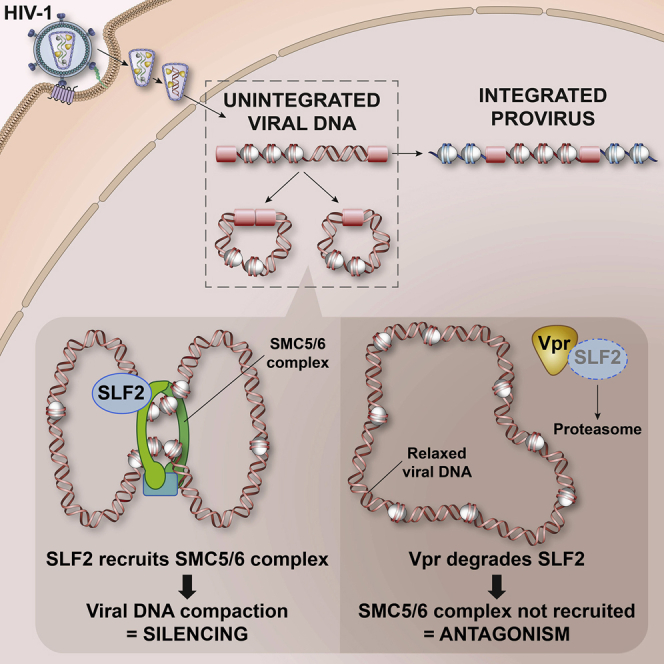

## Teaser

Unlike integrated HIV-1 DNA, extrachromosomal unintegrated HIV-1 DNA is poorly expressed. Dupont et al. show that the host protein SLF2 recruits the SMC5/6 complex to repress unintegrated HIV-1 DNA. This work implicates the SMC5/6 complex in nuclear immunosurveillance of a wide range of extrachromosomal DNAs leading to DNA compaction-based silencing.

## Introduction

Integration of the HIV-1 genome into host chromatin is a hallmark feature of HIV-1 replication, and the epigenetic regulation of integrated lentiviral genomes is extensively studied. However, the linear cDNA produced by reverse transcription gives rise to abundant extrachromosomal viral DNA species, collectively referred to as unintegrated viral DNA. These include linear unintegrated DNA, 1-long terminal repeat (LTR), and 2-LTR circles (Hamid et al., 2017). While unintegrated viral DNAs are generally considered replication incompetent, they are particularly long-lived in non-dividing host cell types such as resting CD4^+^ T cells and macrophages (Gillim-Ross et al., 2005; Pace et al., 2013) and are detected at higher levels in patients receiving HIV-1 integrase inhibitors (Munir et al., 2013), as frequently occurs during highly active antiretroviral therapy (HAART) (Martinez-Picado et al., 2018). Importantly, unintegrated HIV-1 DNA species contain the same genetic and regulatory elements as the integrated provirus and are thus fully capable of gene expression (Wu, 2004). Unintegrated virus is therefore likely to play an underappreciated role in HIV-1 replication by contributing vital gene products in the early stages of infection.

While both integrated and unintegrated reverse transcribed retroviral DNA species are rapidly chromatinized by the host cell, gene expression from unintegrated viral DNA is markedly reduced compared with integrated viral DNA (Geis and Goff, 2019; Sakai et al., 1993). We previously identified a role for the human silencing hub (HUSH) complex in the heterochromatinization and silencing of newly integrated lentiviruses (Tchasovnikarova et al., 2015), and HUSH was subsequently shown to be recruited, via the NP220 protein, to silence unintegrated murine retroviral DNA (Zhu et al., 2018). However, we have been unable to identify a role for HUSH in the silencing of unintegrated primate lentiviruses suggesting the existence of an unrecognized host restriction/silencing pathway acting specifically on extrachromosomal viral DNA.

Previous studies have shown that the HIV-1 accessory protein Vpr enhances gene expression from unintegrated HIV-1 genomes (Poon and Chen, 2003; Poon et al., 2007), yet no underlying mechanism has been identified. We therefore hypothesized that Vpr antagonizes an unknown mechanism for silencing gene expression from unintegrated HIV-1 genomes. Vpr recruits the host Cul4A-DDB1 cullin-RING E3 ubiquitin ligase, via the adaptor protein DCAF1 (CRL4^DCAF1^), to induce the proteasome-mediated degradation of its target cellular proteins (DeHart and Planelles, 2008; Le Rouzic et al., 2007). We recently defined novel targets of the HIV-1 accessory proteins using unbiased mass-spectrometry-based approaches (Greenwood et al., 2016, 2019; Matheson et al., 2015). In contrast to the limited set of host proteins targeted by the other HIV accessory proteins, we found that Vpr induces a global remodeling of the cellular proteome, affecting a wide variety of biological pathways (Greenwood et al., 2019), one of which could be responsible for the silencing of unintegrated virus.

To identify the Vpr-specific substrate(s) responsible for restriction of unintegrated lentiviral gene expression, we performed a sub-genomic CRISPR-Cas9 knockout screen focused on Vpr targets and identified SMC5-SMC6 complex localization factor 2 (SLF2) as required for the restriction of gene expression from unintegrated HIV-1. We characterize a silencing mechanism in which the SMC5/6 complex is recruited to unintegrated lentiviral genomes in an SLF2-dependent manner. By compacting viral chromatin, the SMC5/6 complex creates a repressive chromatin structure which therefore silences viral gene expression. By degrading SLF2, HIV-1 Vpr prevents recruitment of the SMC5/6 complex to unintegrated viral genomes and antagonizes this silencing.

## Results

### HIV-1 Vpr increases unintegrated virus gene expression via CRL4^DCAF1^

**Figure 1:**
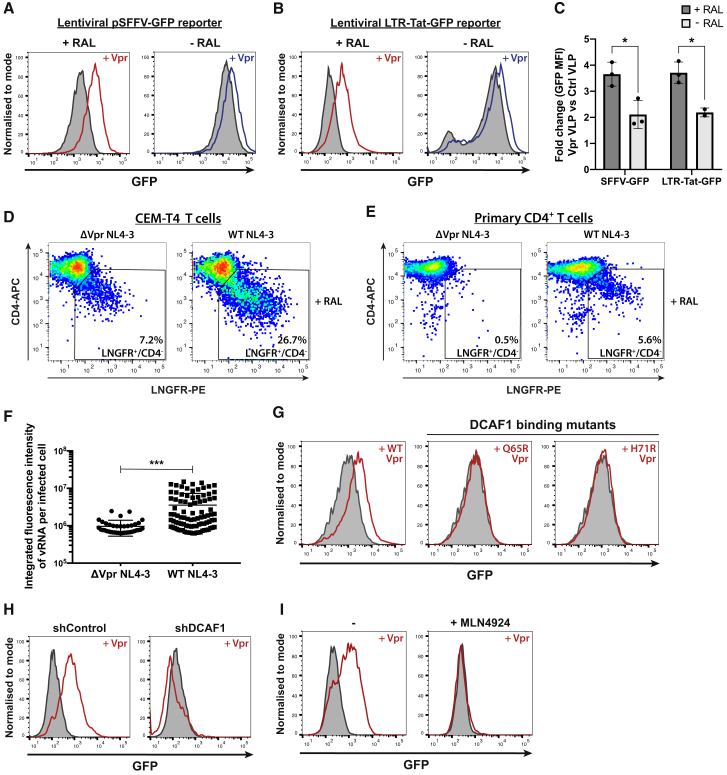
HIV-1 Vpr increases gene expression from unintegrated lentiviral reporters in a cullin-RING E3 ligase dependent manner (A–C) Unintegrated or integrated virus reporter assay. CEM-T4 T cells were co-infected with either SFFV-GFP (A) or LTR-Tat-GFP (B) lentiviral reporters and control (gray shaded) or Vpr-containing (red/blue line) VLPs ± raltegravir (RAL) treatment. GFP expression was evaluated by flow cytometry 72 h post-infection (hpi), representative example (n = 3). Quantified in (C) as the fold change in GFP mean fluorescence intensity (MFI) upon addition of Vpr versus control VLPs. (D and E) Unintegrated ΔVpr NL4-3^LNGFR^ has reduced gene expression. CEM-T4 T cells (D) or primary CD4^+^ T cells (E) were infected with WT or ΔVpr NL4-3^LNGFR^ at equal MOI in the presence of RAL. Cells were stained with α-LNGFR and α-CD4 antibodies 48 hpi and analyzed by flow cytometry (n = 2). (F) Unintegrated ΔVpr NL4-3^GFP^ produces less vRNA. Jurkat T cells were infected with WT or ΔVpr NL4-3^GFP^ in presence of RAL. 48 hpi, vRNA was detected by *in situ* hybridization. Scatter plot shows total vRNA fluorescence per infected cell for 500 cells/condition, filtered for cells with signal intensity ≥2xSD above background. Representative example (n = 2). (G–I) Inhibition of CRL4^DCAF1^ activity abrogates Vpr phenotype. Unintegrated virus reporter assay with SFFV-GFP lentiviral reporters and control or Vpr VLPs upon: Vpr Q65R or H71R point mutation (G), shRNA knockdown of DCAF1 (H), or MLN4924 chemical cullin inhibition (I). Representative histograms (n = 2). ^∗^p < 0.05; ^∗∗∗^p < 0.001. Error bars show standard deviation. See also Figure S1.

To show that HIV-1 Vpr enhances gene expression from unintegrated virus, we infected CEM-T4 cells with lentiviral reporters expressing GFP from either the spleen focus-forming virus promoter (SFFV) or the HIV-1 LTR (Figures S1A and S1B). Vpr protein was delivered in virus-like particles (VLPs) in the presence or absence of the viral integrase inhibitor raltegravir. Vpr significantly enhanced GFP expression from both lentiviral reporters (Figures 1A–1C), most markedly when integration was inhibited by raltegravir, regardless of whether the Vpr was delivered in VLPs (Figures 1A–1C) or inside reporter virions (Figure S1E). When Vpr VLPs were added to cells already containing stably integrated virus, no increase in gene expression was seen (Figure S1F, right column), confirming the specificity of this effect for unintegrated viral genomes. We replicated our observation using a full-length NL4-3 reporter virus in which a low-affinity nerve growth factor receptor (LNGFR) reporter has been inserted downstream of the *nef* gene and separated by a self-cleaving P2A peptide, therefore providing a surrogate cell surface marker for Nef protein expression (Naamati et al., 2019) (Figure S1C). In the presence of raltegravir, reduced Nef expression in the Vpr-deletion mutant virus, as determined by decreased cell surface LNGFR, correlated with reduced depletion of cell surface CD4 both in CEM-T4 cells (Figure 1D) and in primary CD4^+^ T cells (Figure 1E). In the absence of raltegravir, Vpr deletion had minimal effect (Figures S1H and S1I). Furthermore, in the presence of raltegravir, infection with Vpr-deletion mutant viruses showed decreased HIV-1 RNA levels, as detected by *in situ* hybridization, compared with wild-type (WT) virus (Figures 1F and S1D). Our results therefore confirm that HIV-1 Vpr enhances viral gene expression from unintegrated viral genomes (Poon and Chen, 2003; Poon et al., 2007).

We hypothesized that Vpr antagonizes a silencing mechanism specific for unintegrated HIV-1 genomes, through degradation of an unknown host repressive factor. This implies a dependency of Vpr on host protein degradation via the CRL4^DCAF1^ ubiquitin E3 ligase. Indeed, two Vpr point mutants, which are unable to bind the ligase adaptor protein DCAF1, Q65R Vpr (Le Rouzic et al., 2007), and H71R Vpr (Hrecka et al., 2007), failed to increase unintegrated virus expression (Figure 1G). Furthermore, shRNA-mediated depletion of DCAF1 (Figures 1H and S1G) or chemical inhibition of cullin E3 ligase activity using the neddylation inhibitor MLN4924 (Figure 1I) also abrogated the enhanced effect of WT Vpr on unintegrated virus gene expression. Therefore, CRL4^DCAF1^ activity is required for Vpr to relieve silencing of unintegrated virus expression, supporting a role for host factor degradation.

### A sub-genomic CRISPR-Cas9 library screen implicates SMC5-SMC6 complex localization factor 2 in the silencing of unintegrated HIV-1

**Figure 2:**
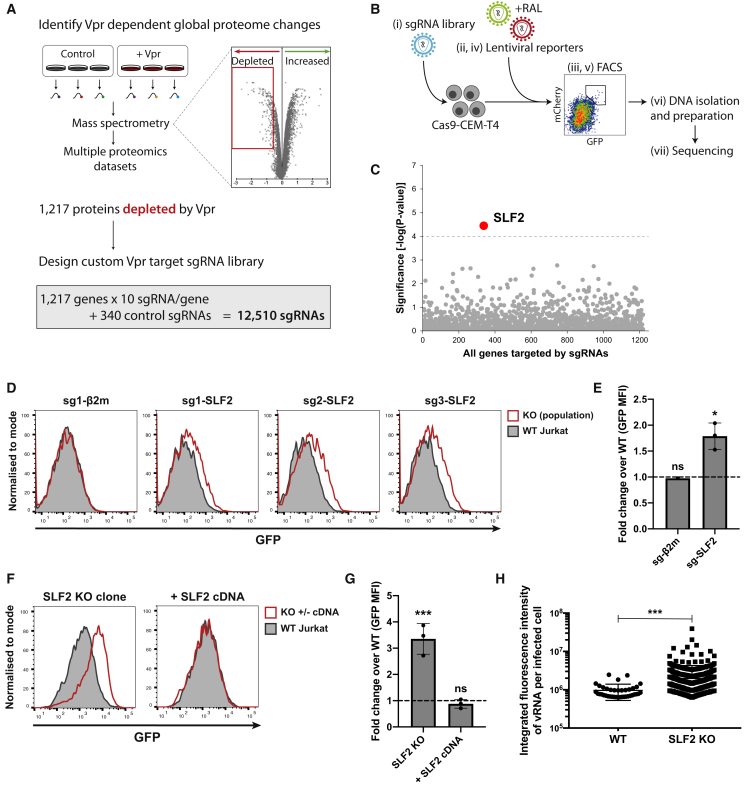
Targeted CRISPR-Cas9 knockout screen implicates SMC5/6 complex localization factor 2 in silencing of unintegrated virus (A–C) CRISPR-Cas9 knockout screen for unintegrated virus silencing factors. (A) A custom sgRNA library was constructed, containing sgRNAs targeting genes encoding Vpr-depleted proteins identified from existing proteomics datasets and used for a CRISPR-Cas9 knockout screen outlined in (B). A pooled Vpr library knockout population (Bi) was infected with GFP and mCherry-lentiviral reporters in presence of RAL (Bii). Rare high expressing cells were enriched by FACS (Biii) followed by repeated reporter virus infection (Biv) and sorting (Bv). DNA was isolated from sorted and unsorted library populations (Bvi) and prepared for next-generation sequencing (Bvii). (C) Candidate genes essential for unintegrated virus silencing were identified using MAGeCK. Genes scoring above multiple-testing-corrected threshold are highlighted. (D–G) Validation of screen hit. Unintegrated virus reporter infection of mixed knockout (KO) populations 7 days post-sgRNA transduction of Cas9-Jurkat. Flow cytometry 72 hpi (D), quantified as fold change GFP MFI over WT Jurkat (E). Representative example (n = 3). (F) Unintegrated virus reporter infection of clonal SLF2 KO cell line ± full-length SLF2 cDNA complementation, data from n = 3 quantified in (G). (H) Unintegrated Vpr-deletion NL4-3 reporters produce more vRNA upon SLF2 KO. WT or SLF2 KO Jurkat T cells were infected with ΔVpr NL4-3^GFP^ in presence of RAL. 48 hpi, viral RNA was detected by *in situ* hybridization and quantified as previously described. Data are representative example of n = 2. Error bars show standard deviation. ns, p > 0.05; ^∗^p < 0.05; ^∗∗∗^p < 0.001. See also Figures S2–S4.

We recently showed that Vpr orchestrates a systems-level remodeling of the host cell proteome (Greenwood et al., 2019). The extensive list of >1,200 putative Vpr targets from our proteomic datasets allowed us to take a forward genetics approach to identify the critical Vpr target responsible for silencing unintegrated virus. Our rationale was that if Vpr-targeted degradation of a host protein increases lentiviral expression, this effect should be phenocopied using CRISPR-Cas9-mediated gene knockouts. Our proteomic datasets identified 1,217 protein targets, which are depleted in a Vpr-dependent manner (Greenwood et al., 2019). We therefore cloned a sub-genomic sgRNA library of these Vpr targets, containing 10 independent sgRNAs per gene, as well as 340 control sgRNAs (Figure 2A; Table S1). Our screen for host factors, which repress unintegrated virus gene expression was thereby focused on cellular genes encoding putative Vpr targets. The screen was initiated by transducing Cas9-CEM-T4 cells with the Vpr target sgRNA library to generate a pooled population of knockout cells and subsequently co-infecting these mutagenized cells with GFP- and mCherry-lentiviral reporters, in the presence of raltegravir. We used fluorescence-activated cell sorting (FACS) to select a population of GFP^high^/mCherry^high^ cells (Figure 2B). This enriched population underwent a second round of reporter infection and FACS to further select rare mutant cells with increased expression of unintegrated virus. The sgRNAs from the sorted population, together with the unsorted pooled knockout library population were submitted for next-generation sequencing. Using the MAGeCK algorithm, we identified SMC5-SMC6 complex localization factor 2 (*SLF2*, previously known as *FAM178A*) as the only significant hit from the screen and therefore the putative Vpr target potentially responsible for inhibiting host restriction of unintegrated virus (Figure 2C).

To validate a role for SLF2 in restricting unintegrated virus expression, we generated pooled CRISPR-Cas9 SLF2 knockout populations in Cas9-Jurkat T cells. Unintegrated reporter virus expression was increased for all three independent SLF2 sgRNAs but not for a β2-microglobulin (β2m) control sgRNA (Figures 2D and 2E). Despite the classification of SLF2 as an essential gene (Blomen et al., 2015), an extensive cloning effort identified a single Jurkat T cell SLF2 knockout clone (Figures S2A and S2B), which showed significantly increased unintegrated reporter expression compared with WT cells (Figures 2F and 2G). The loss of silencing in the SLF2 knockout clone was fully restored following complementation with full-length SLF2 cDNA (Figures 2F and 2G). We also examined viral gene expression from HIV-1 NL4-3^LNGFR^ reporter viruses and confirmed that SLF2 knockout increased gene expression from unintegrated Vpr deletion (Figure S3A) but not WT viruses (Figure S3B). Furthermore, *in situ* hybridization showed increased viral RNA levels in the SLF2 knockout clone in the presence of raltegravir (Figure 2H). These data imply that targeted deletion of SLF2 compensated for the loss of Vpr to increase gene expression in the Vpr-deletion mutant virus from unintegrated NL4-3 reporter viruses.

### Unintegrated virus gene expression is restricted by the SMC5/6 complex in an SLF2-dependent but SLF1-independent manner

**Figure 3:**
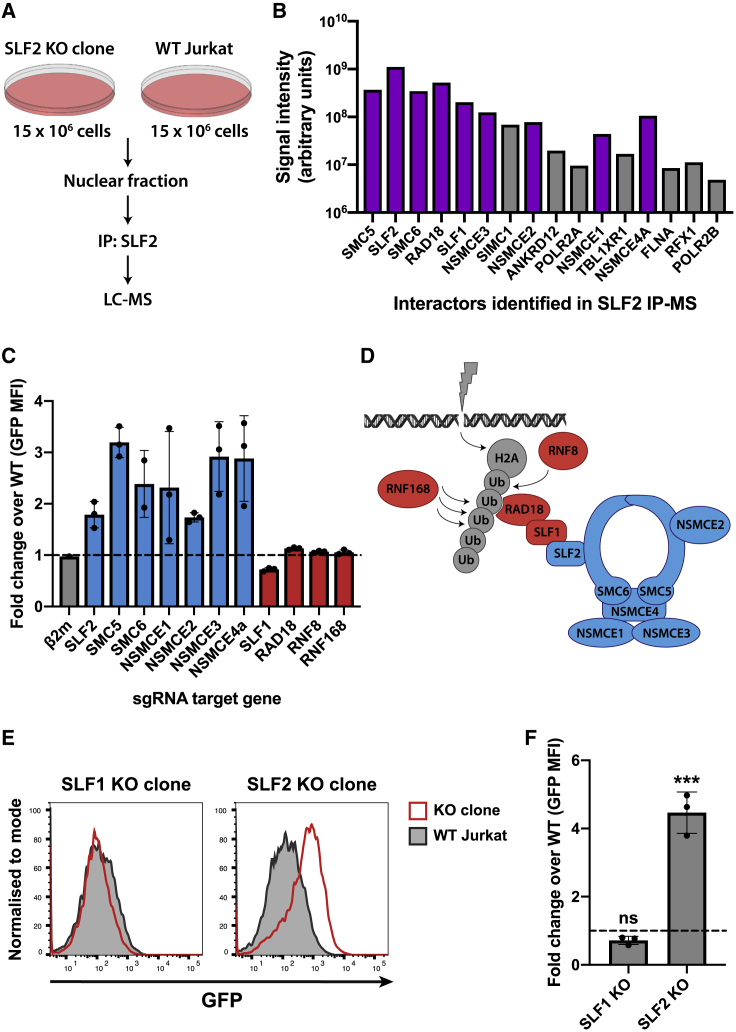
Unintegrated lentivirus expression is restricted by the SMC5/6 complex in an SLF2-dependent but SLF1-independent manner (A and B) IP-MS identifies SMC5/6 complex components as SLF2-interactors. (A) Immunoprecipitated material from endogenous SLF2 IP in SLF2-KO and WT Jurkat cells was analyzed by mass spectrometry. Interactors displayed in (B) satisfy inclusion criteria of being (1) undetected in IP from SLF2-KO cells and (2) detected with ≥3 peptides in IP from WT cells. Interactors are displayed in descending order by number of unique peptides identified. Representative dataset (n = 2). Purple bars indicate SLF2/SMC6 interactors identified by (Räschle et al., 2015) (C–F) Knockout of SMC5/6 complex but not SLF1 increases unintegrated virus expression. Unintegrated virus reporter infection of mixed KO populations 7 days post-sgRNA transduction of Cas9-Jurkat. Flow cytometry 72 hpi quantified as fold change GFP MFI over WT (C). Each bar represents data for 3 independent sgRNAs (n = 3). (D) Schematic of DNA damage recruitment of SMC5/6 complex proposed by (Räschle et al., 2015); Blue, KO increases unintegrated virus expression; red, no phenotype. (E) Unintegrated virus reporter infection of clonal SLF1 and SLF2-KO cell lines, flow cytometry 48 hpi. Data from n = 3 quantified in (F). Error bars show standard deviation. ns, p > 0.05; ^∗∗∗^p < 0.001. See also Figures S2 and S4.

SLF2 is a poorly characterized protein but was recently implicated in the recruitment of the SMC5/6 complex to sites of DNA damage (Räschle et al., 2015). We took a proteomic approach to identify proteins that might act with SLF2 to silence unintegrated virus. Mass spectrometric analysis of endogenous SLF2 immunoprecipitated from WT versus SLF2-knockout Jurkat T cell nuclei identified a short list of SLF2-interacting proteins (Figures 3A and 3B). This included all known components of the human SMC5/6 complex (SMC5, SMC6, and NSMCE1-4A) as well as the RAD18, SLF1, SLF2 proteins, in agreement with the previous study examining SLF2 under DNA damage conditions (Räschle et al., 2015).

The SMC5/6 complex is an enigmatic host protein complex implicated in DNA repair and maintenance of genome integrity (Aragón, 2018). To determine whether the SMC5/6 complex plays a role in restricting unintegrated lentiviral gene expression, we generated CRISPR-Cas9 knockout Jurkat T cell lines, individually depleted of SLF2, all six SMC5/6 complex core components (SMC5, SMC6, and NSMCE1-4A) as well as the 4 genes (SLF1, RAD18, RNF8, and RNF168) implicated in DNA-damage-induced recruitment and ubiquitin scaffold formation (Mailand et al., 2007; Panier et al., 2012; Räschle et al., 2015). Targeting of SLF2 and each individual core SMC5/6 complex component with three independent sgRNAs increased unintegrated virus expression in the pooled knockout populations compared with WT cells (Figures 3C and 3D). In contrast, targeted depletion of gene products reported to act “upstream” of SLF2 for recruitment of the SMC5/6 complex to sites of DNA damage (SLF1, RAD18, RNF8, and RNF168; see Figure 3D) had no effect on unintegrated viral gene expression. Other than SLF2, none of the core SMC5/6 complex components were depleted by Vpr in our proteomic analysis (Greenwood et al., 2019) and were not, therefore, included in the Vpr library used in the genetic screen. This library did, however, contain sgRNAs targeting SLF1, RNF8, and RNF168, and their lack of unintegrated virus expression phenotype agrees with them not being hits in our screen.

Our data imply that the core components of the SMC5/6 complex are required for both viral restriction and DNA damage repair. Moreover, as SLF2 recruits the SMC5/6 complex to sites of exogenous DNA damage via SLF1 (Figure 3D) (Räschle et al., 2015), and neither SLF1 nor the “upstream” components (SLF1, RAD18, RNF8, and RNF168) are required for viral restriction, we suggest that recruitment of the SMC5/6 complex to restrict viral expression is independent of its role in DNA damage/repair. These observations were further corroborated by isolation of an SLF1-knockout clone (Figures S2C and S2D), which, like the pooled knockout population, showed no increase in unintegrated virus expression (Figures 3E and 3F). We therefore propose that unintegrated virus gene expression is restricted by the SMC5/6 complex in an SLF2-dependent, SLF1-independent pathway that is separate from its role in DNA repair.

### The N terminus of SLF2 is dispensable for interaction with the SMC5/6 complex and restriction of unintegrated virus

The yeast SMC5/6 complex is better characterized than its mammalian counterpart, and a distant protein homology search identified the Smc5-Smc6-associated factor Nse6 as the *S. pombe* ortholog of SLF2 (Figure S4A), as previously proposed (Räschle et al., 2015). The yeast Nse6 protein (522 aa) aligns exclusively to the C terminus of SLF2 and is much shorter than the 1,173 residue human SLF2 protein (Figure S4A), suggesting that the intrinsically disordered ∼650 N-terminal amino acids of SLF2 (Figure S4B) are a more recent acquisition. We examined whether an HA-tagged, truncated minimal SLF2(590–1,173) could complement the SLF2 KO clone. This minimal SLF2 not only complemented the clonal SLF2 knockout cell line, but silencing was more potent than full-length SLF2 (Figure S4C). Moreover, this minimal SLF2 co-immunoprecipitated the entire SMC5/6 complex (Figures S4D and S4E), suggesting the N terminus of SLF2 is dispensable for unintegrated virus restriction and may serve a regulatory function.

### Vpr associates with and selectively degrades SLF2 to antagonize silencing of unintegrated virus by the SMC5/6 complex

**Figure 4:**
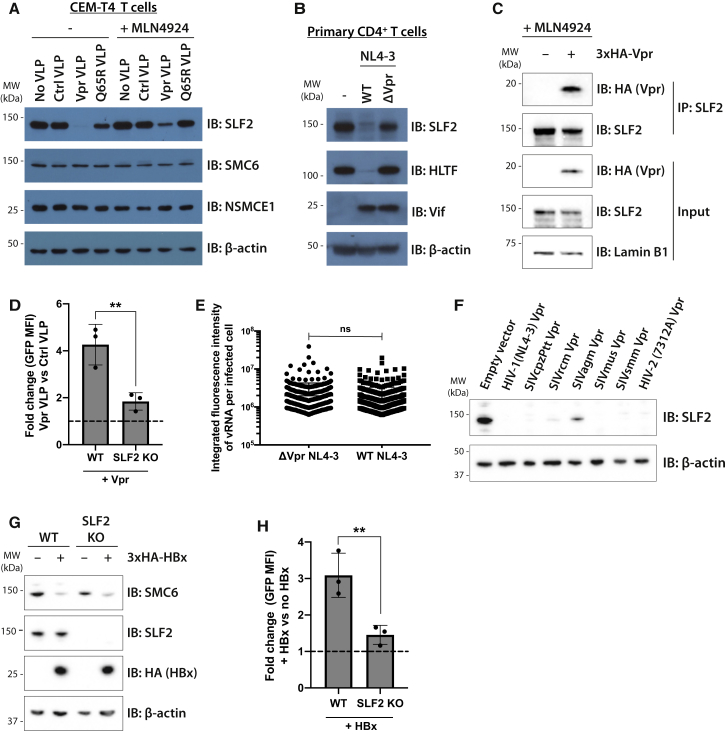
Unintegrated lentivirus restriction by the SMC5/6 complex is antagonized by viral accessory proteins Vpr and HBx (A and B) Endogenous SLF2 is depleted by HIV-1 Vpr. (A) CEM-T4 T cells were transduced with control VLP, Vpr VLP, or Q65R Vpr VLP, with or without 1-μM MLN4924. Cell lysates were harvested 24 hpi and analyzed by immunoblotting. (B) Primary CD4^+^ T cells were infected with WT or ΔVpr NL4-3^LNGFR^. 48 hpi, infected cells were enriched by AFMACS and lysates analyzed by immunoblotting. (C) SLF2 interacts with 3xHA-Vpr. CEM-T4 T cells were preincubated with 1-μM MLN4924 and transduced with 3xHA-Vpr. 24 hpi, nuclear extracts were immunoprecipitated with an SLF2 antibody and analyzed by immunoblotting. (D) SLF2 KO reduces Vpr effect on unintegrated virus expression. Unintegrated virus reporter assay in WT or clonal SLF2 KO cells co-transduced with control or Vpr VLPs. Flow cytometry 48 hpi (n = 3), quantified as fold change in GFP MFI upon addition of Vpr versus control VLPs. (E) vRNA levels are unchanged upon Vpr deletion in SLF2 KO cells. Clonal SLF2 KO cells were infected with WT or ΔVpr NL4-3^GFP^ reporters in presence of RAL. vRNA was detected by *in situ* hybridization 48 hpi and quantified as previously described. Representative data of n = 2. (F) Vpr ability to degrade SLF2 is evolutionarily conserved. Jurkat T cells were transduced with primate lentiviral Vpr constructs, and Vpr-expressing cells isolated by GFP^+^ FACS 48 hpi followed by immunoblotting of lysates. Representative blot (n = 2). (G and H) HBV HBx rescues gene expression from unintegrated lentiviral reporters. (G) WT or SLF2 KO Jurkat cells were transduced with 3xHA-HBx, puromycin selected, and lysates analyzed 96 hpi by immunoblotting. Representative blot (n = 3). (H) Unintegrated virus reporter infection of WT and SLF2 KO cells ± 3xHA-HBx, flow cytometry 72 hpi. Data quantified as fold change in GFP MFI upon addition of HBx (n = 3). Error bars show standard deviation. ns, p > 0.05; ^∗∗^p < 0.01. See also Figure S5.

The inclusion of SLF2 in our Vpr target library was based on its Vpr-mediated depletion, as reported in our previous proteomics datasets (Greenwood et al., 2019). These data suggested SLF2 as a genuine Vpr target and were strengthened by showing CRL4^DCAF1^-dependent depletion of SLF2 by Vpr. Immunoblotting of cells transduced with VLPs containing WT Vpr, but not the Q65R Vpr mutant, which is unable to bind DCAF1, showed a complete loss of SLF2 (Figure 4A), which was rescued by pan-cullin inhibition with MLN4924. Importantly, levels of SMC6 and NSMCE1, two core SMC5/6 complex components, were unaffected by Vpr. SLF2 was also depleted by WT but not Vpr-deletion NL4-3^LNGFR^ viruses in infected primary CD4^+^ T cells (Figures 4B and S5A). SLF2 is likely to be a direct Vpr target as, in the presence of MLN4924, endogenous SLF2 co-immunoprecipitates 3xHA-tagged Vpr (HA-Vpr) in CEM-T4 T cells (Figure 4C) and in the reverse immunoprecipitation HA-Vpr co-immunoprecipitates endogenous SLF2 (Figure S5B). Therefore, Vpr selectively interacts with and degrades SLF2 to antagonize silencing of unintegrated virus by the SMC5/6 complex. Moreover, the ability of Vpr to increase gene expression from unintegrated viral reporters was significantly reduced in SLF2-knockout cells (Figure 4D), and the absence of SLF2 also abrogated the effect of Vpr deletion on viral RNA expression from full-length NL4-3 reporters (Figure 4E). Unintegrated virus gene expression in the SLF2-knockout clone was therefore Vpr insensitive, in contrast to the parental WT Jurkat cell line. The absence of SLF2 did not affect the well-established ability of Vpr to induce cell-cycle arrest (Figure S5C) (Jowett et al., 1995; Re et al., 1995; Rogel et al., 1995), nor did it effect depletion of the known Vpr targets UNG2 (Schröfelbauer et al., 2005), HLTF (Hrecka et al., 2016; Lahouassa et al., 2016), or DCAF1 (Lapek et al., 2017) (Figure S5D). Furthermore, SLF2 IP-MS did not identify any known Vpr targets (Figure 3B), and we therefore find no evidence for a role of SLF2 in previously assigned Vpr functions, other than gene expression from unintegrated virus.

### Primate lentiviral Vpr-mediated depletion of SLF2 is evolutionarily conserved

Viral targeting of host genes involved in viral resistance is typically evolutionarily conserved. To determine whether Vpr’s ability to degrade SLF2 is a conserved function of the Vpr lineage we tested NL4-3 Vpr from HIV-1 together with a panel of lentiviral Vpr proteins from divergent primate lineages including simian immunodeficiency virus (SIV) from chimpanzees (SIVcpzPtt), red-capped mangabeys (SIVrcm), African green monkeys (SIVagm), mustached monkeys (SIVmus), sooty mangabeys (SIVsmm), and a primary HIV-2 isolate. All these Vpr constructs effectively depleted SLF2 from Jurkat cells (Figure 4F) with the exception of SIVagm-derived Vpr, whose poor expression (Figure S5E) likely accounted for its partial degradation of SLF2. This evolutionary conservation of SLF2 depletion by Vpr within the lentiviral lineage is strongly suggestive of a selective *in vivo* advantage of antagonizing silencing by SLF2 depletion.

The SMC5/6 complex was previously proposed as a restriction factor for hepatitis B virus (HBV), which persists as an extrachromosomal covalently closed circular DNA (cccDNA) episomal viral genome, in the nuclei of infected cells (Decorsière et al., 2016; Murphy et al., 2016). Gene expression from HBV cccDNA, like unintegrated HIV-1, is restricted by the host cell yet is enhanced by the HBx viral protein (van Breugel et al., 2012), which degrades SMC5 and SMC6 (Decorsière et al., 2016; Murphy et al., 2016). We therefore tested whether ectopic expression of HBV HBx could substitute for HIV-1 Vpr and rescue gene expression from unintegrated HIV-1 reporters. Immunoblotting confirmed the HBx-dependent depletion of SMC6 from both WT- and SLF2-knockout Jurkat T cells with no effect on SLF2 levels (Figure 4G). Ectopic expression of HBx increased gene expression from unintegrated lentiviral reporters (Figure 4H), an effect that was significantly reduced in the SLF2 knockout clone. These experiments confirm a role for the SMC5/6 complex in SLF2-mediated restriction of unintegrated virus expression. They also provide a novel example of convergent viral evolution, whereby two unrelated viruses (HBV and HIV) use a similar mechanism to prevent the SMC5/6 complex from restricting extrachromosomal, nuclear viral gene expression.

### Silencing of unintegrated HIV-1 gene expression is independent of the HUSH complex and is characterized by depletion of H3K4me3 histone marks

Given the different life cycles of the viruses restricted by the SMC5/6 complex, we focused on a likely common mechanism for regulating gene expression: the chromatin landscape. Restriction of gene expression from unintegrated retroviral DNA has previously been linked to repressive H3K9me3 deposition through recruitment of the HUSH complex to integrase-deficient Moloney murine leukemia virus (MLV) reporters (Zhu et al., 2018). However, we did not observe any increase in unintegrated virus gene expression for our HIV-1-based lentiviral reporters, either in a clonal *TASOR* knockout cell line, or when performing pooled knockouts. Knocking out HUSH complex components (TASOR, MPP8, and PPHLN1) or the DNA-binding protein NP220 proposed to recruit the HUSH complex to unintegrated MLV genomes (Figures S6A and S6B), with three independent sgRNAs per gene had no effect on unintegrated HIV-1 gene expression, contrary to what we observed for knocking out SMC5/6 complex components. Thus, the silencing mechanism we report for unintegrated HIV-1 is independent of HUSH complex activity.

**Figure 5:**
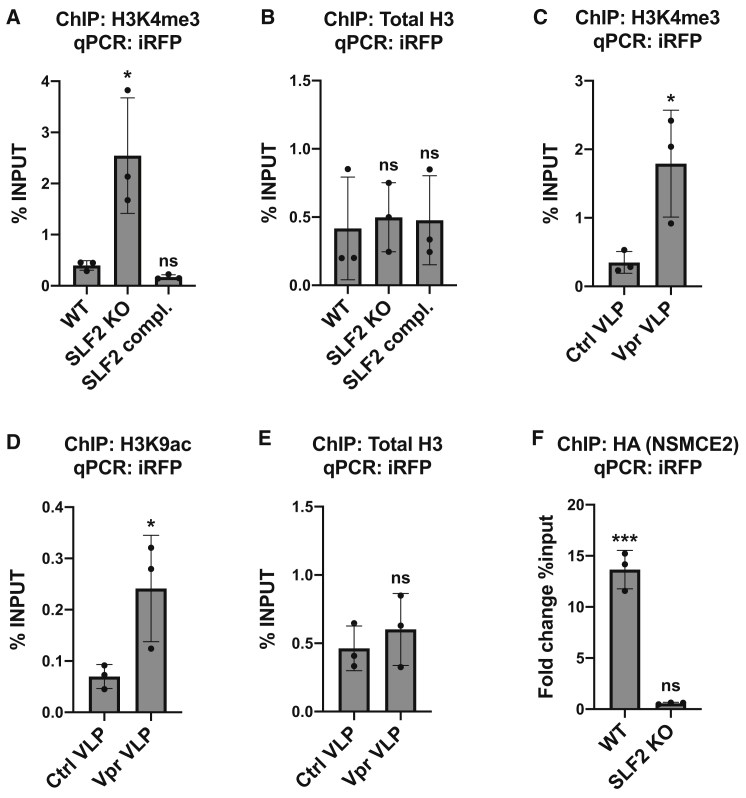
The SMC5/6 complex is recruited in an SLF2-dependent manner to unintegrated lentiviral genomes leading to a loss of H3K4me3 and H3K9ac on viral chromatin (A and B) Knockout of SLF2 increases H3K4me3 on unintegrated virus. WT, clonal SLF2 KO and SLF2 complemented SLF2 KO cells were infected with SFFV-iRFP lentiviral reporters in presence of RAL. 48 hpi, ChIP was performed using antibodies against (A) H3K4me3 and (B) total H3. qPCR data from each ChIP experiment were calculated as the percentage of input DNA. Histograms summarize data from n = 3 experiments. (C–E) Vpr-mediated depletion of SLF2 increases H3K4me3 and H3K9ac on unintegrated virus. WT cells were co-transduced with iRFP reporters and either control or Vpr VLPs in presence of RAL. 48 hpi, ChIP was performed using antibodies against (C) H3K4me3, (D) H3K9ac, and (E) total H3, calculated as the percentage of input DNA (n = 3). (F) The SMC5/6 complex binds unintegrated viral genomes via SLF2. WT or SLF2 KO cells expressing 3xHA-NSMCE2 were infected with SFFV-iRFP in presence of RAL. 48 hpi, ChIP was performed using antibodies against the HA-tag. Data were calculated as the fold change in percentage of input DNA compared with a matched control ChIP experiment performed in reporter-infected WT or SLF2 cells that did not express HA-NSMCE2 (n = 3). Error bars show standard deviation. ns, p > 0.05; ^∗^p < 0.05; ^∗∗∗^p < 0.001. See also Figure S6.

Similar to MLV, unintegrated HIV-1 DNA species are rapidly chromatinized with increased levels of the histone modification H3K9me3, characteristic of heterochromatin (Geis and Goff, 2019; Wang et al., 2016). To determine whether the SMC5/6 complex orchestrates histone-methylation-dependent heterochromatin formation on unintegrated virus, we performed chromatin immunoprecipitation (ChIP) on cells infected with the iRFP reporter virus. We found no change in the two well-characterized histone silencing marks, H3K9me3 and H3K27me3, on unintegrated viral genomes following SLF2 knockout (Figures S6C and S6D). Conversely, levels of the histone mark H3K4me3, characteristic of active gene transcription, were significantly increased on unintegrated virus in SLF2 knockout cells (Figure 5A) with no change in total H3 levels (Figure 5B). Similarly, Vpr-mediated depletion of SLF2 led to increased levels of both H3K4me3 (Figure 5C) and the activating histone mark H3K9ac (Figure 5D) with no change in total H3 levels (Figure 5E). Taken together, our data suggest that the SMC5/6 complex establishes a repressive chromatin environment on unintegrated viral genomes via a mechanism independent of conventional heterochromatin formation.

### The SMC5/6 complex binds to unintegrated HIV-1 genomes via SLF2 and induces silencing by compacting viral chromatin

To determine whether the SMC5/6 complex is directly recruited to viral DNA, we undertook a ChIP analysis of unintegrated virus in cells expressing HA-tagged SMC5/6 complex components. Multiple core components of the SMC5/6 complex were HA tagged, but only expression of 3xHA-NSMCE2 (HA-NSMCE2) could be detected. Consequently, we infected cells with the iRFP reporter virus in the presence or absence of HA-NSMCE2. HA-NSMCE2 ChIP-PCR analysis showed a significant enrichment of HA-NSMCE2 to viral genomes in WT but not in SLF2-knockout cells (Figure 5F). The SMC5/6 complex is therefore recruited to unintegrated lentivirus in an SLF2-dependent manner.

**Figure 6:**
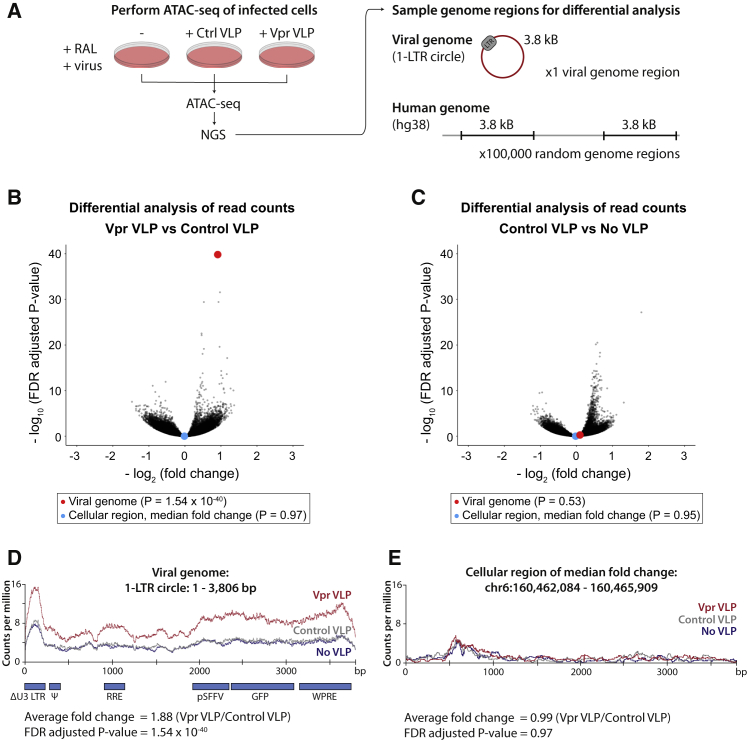
The SMC5/6 complex induces compaction of unintegrated lentiviral genomes (A–E) ATAC-seq shows increased unintegrated virus chromatin accessibility upon Vpr-mediated SLF2 depletion. (A) WT Jurkat cells were co-infected with a GFP reporter lentivirus and control or Vpr VLPs in the presence of RAL, and ATAC-seq performed 48 hpi. Reads were aligned to the human and viral 1-LTR circle reference genomes. To quantify effects on chromatin accessibility, 100,000 cellular genome regions of equal size to the viral genome (3.8 kB) were randomly defined, and the fold change in normalized sequence coverage between conditions calculated for each region, viral or cellular. For each data point, significance was determined using Fisher’s t test, adjusted by FDR to correct for multiple testing. Comparisons were visualized as volcano plots summarizing data for all 100,000 cellular regions and the viral genome (plotting regions with >50 aligned reads): (B) comparing data for Vpr VLP with control VLP and (C) comparing data for control VLPs with No VLPs. The viral genome (red) and the cellular region of median fold change (blue) are highlighted as enlarged data points for each comparison. Normalized read density for the viral genome is displayed in (D) and for a representative cellular region of median fold change in (E). See also Figure S7.

The key characteristic of all SMC protein complexes is their ability to topologically entrap and translocate DNA via their ATPase domains (Hassler et al., 2018). For the well-studied SMC complexes, cohesin (SMC1/3) and condensin (SMC2/4), DNA translocation and ATP hydrolysis are linked to loop extrusion and chromatin compaction (Kim et al., 2019; Terakawa et al., 2017). Given the high structural similarity between members of the SMC family, we hypothesized that the SMC5/6 complex might act in a similar manner to compact chromatin. Formation of a repressive chromatin environment would therefore result from a direct physical effect of the SMC5/6 complex on chromatin. To determine whether recruitment of SLF2 and the SMC5/6 complex affects viral chromatin compaction, we used an assay for transposase-accessible chromatin using sequencing (ATAC-seq), which exploits a Tn5 transposase to probe chromatin accessibility (Buenrostro et al., 2013). If a chromatin region is accessible, Tn5 will fragment the genome in that location, which is identified by next-generation sequencing of released chromatin fragments. We focused our analysis on Vpr-mediated SLF2 depletion by performing ATAC-seq on WT Jurkat cells co-transduced with a lentiviral reporter and either Vpr VLPs or control VLPs in the presence of raltegravir. The ATAC-seq experiment was technically successful with >110 × 10^6^ sequencing reads mapped to the human genome and 19,000–43,000 reads mapped to the unintegrated viral genome (Figures S7A–S7C). To assay the effect of Vpr on viral and cellular chromatin accessibility, we performed a differential analysis of ATAC-seq signal across the unintegrated lentiviral reporter (using a 1-LTR circle reference genome) as well as 100,000 randomly selected cellular genome regions of equal length (3.8 kB) (Figure 6A). For each region, viral or cellular, we determined the fold change in normalized read counts across the region and calculated the associated statistical significance. Vpr VLPs caused a highly significant (1.9-fold) increase in chromatin accessibility for the viral genome compared with control VLPs (Figure 6B, red dot), an effect which was observed across the entire length of the viral genome (Figure 6D). The addition of control VLPs had no effect on viral chromatin accessibility (Figures 6C and 6D). Moreover, this Vpr-dependent increase of chromatin accessibility was not seen globally across the 100,000 randomly sampled 3.8-kB regions of the cellular genome (Figure 6B) as illustrated by the cellular region that had the median fold change (Figures 6B, blue dot, and 6E). The addition of Vpr and subsequent SLF2 depletion therefore specifically increased chromatin accessibility for the viral genome and had no global effect on the cellular genome. This is consistent with Vpr driving decompaction of unintegrated viral chromatin to antagonize silencing by the SMC5/6 complex and increase gene expression from extrachromosomal viral DNA species.

**Figure 7:**
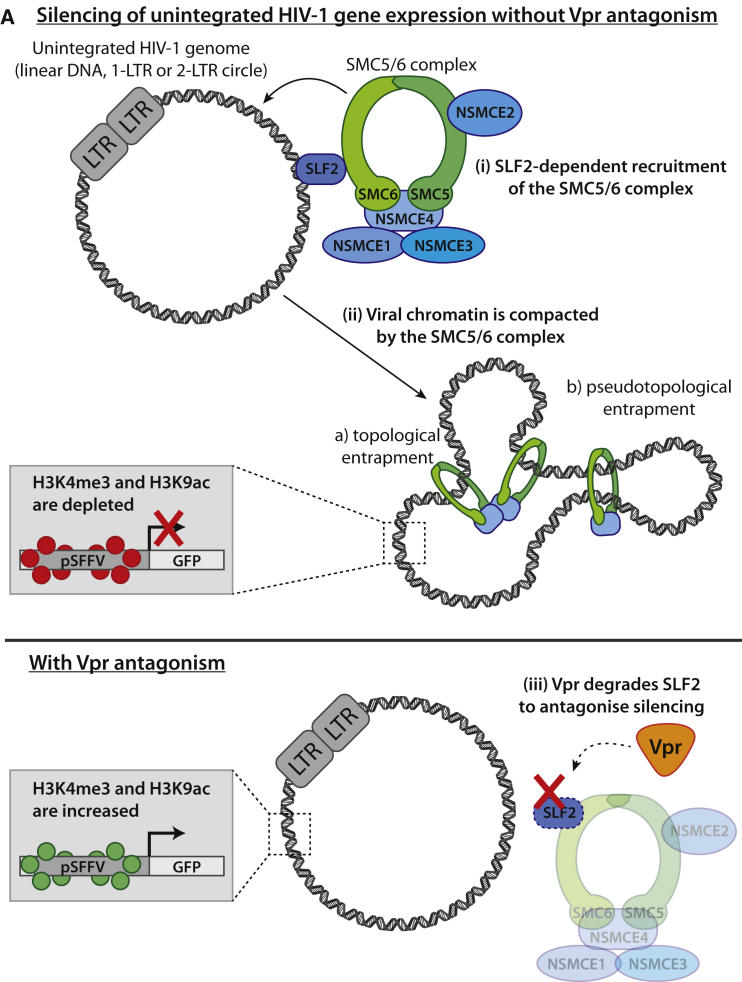
Model for silencing of unintegrated HIV-1 by the SMC5/6 complex (A) Schematic of model: (Ai) in the absence of antagonism by Vpr, the SMC5/6 complex is recruited to unintegrated HIV-1 genomes in an SLF2-dependent manner. (Aii) This leads to compaction of viral chromatin by either topological or pseudotopological entrapment of DNA. A repressive chromatin environment is created, and viral gene expression is silenced. (Aiii) HIV-Vpr degrades SLF2 to antagonize restriction by the SMC5/6 complex, thereby rescuing gene expression from unintegrated viral genomes.

In conclusion, we propose a model in which SLF2-dependent recruitment of the SMC5/6 complex to unintegrated HIV-1 genomes leads to chromatin compaction and loss of activating histone marks, creating a repressive chromatin structure with concomitant silencing of viral gene expression (Figure 7A). This silencing is antagonized by lentiviral Vpr, which degrades the SMC5/6 complex recruitment factor SLF2 via CRL4^DCAF1^, thereby rescuing gene expression from extrachromosomal HIV-1 DNA species.

## Discussion

Unintegrated HIV-1 DNA species contain the same genetic and regulatory elements as the integrated provirus and are fully capable of gene expression (Wu, 2004). Why unintegrated HIV-1 genomes are so poorly expressed has, therefore, remained unclear. Here, we implicate a central role for SLF2 in recruiting the SMC5/6 complex to compact and silence the viral DNA. We show SLF2-dependent recruitment of the SMC5/6 complex to unintegrated viral genomes, which leads to repression of gene expression through compaction of viral chromatin and loss of active histone marks. Lentiviral Vpr associates with and degrades SLF2 in a CRL4^DCAF1^-dependent manner, thereby antagonizing this silencing.

Enhanced gene expression from unintegrated HIV-1 DNA is seen with both virion-packaged Vpr or VLP-delivered Vpr and occurs in primary human CD4^+^ T cells as well as cell lines. The raltegravir-independent, Vpr-mediated increase in viral gene expression is also likely due to the de-repression of unintegrated genomes, as this phenotype was not seen with stably integrated virus. In the context of natural infection, the abundant unintegrated viral DNA species are therefore not simply “dead-end” products. They provide an additional source of viral gene expression, which is enhanced by Vpr. At early time points post-infection, gene expression from unintegrated virus may therefore contribute to the success of the integrated viral genome and form the basis for a productive infection—particularly if the virus integrates into a site that is not well transcribed.

As the only accessory protein to be delivered with the incoming virion into the infected cell, Vpr is responsible for antagonizing early host restriction and preparing the cell for viral replication prior to integration. Our recent studies show that Vpr globally remodels the host cell proteome, targeting multiple cellular proteins for proteasomal degradation via the CRL4^DCAF1^ E3 ligase complex (Greenwood et al., 2019). We therefore find it neither helpful nor necessary to consider a “prime” role for Vpr as it clearly has multiple effects on cellular phenotypes and viral replication. Vpr originated in the SIV primate lentiviral lineage and its antagonism of SLF2 is evolutionarily conserved across a diverse range of primate lentiviral lineages, highlighting the importance of antagonizing restriction of extrachromosomal lentiviral gene expression. Antagonism of SMC5/6 by HBx is similarly conserved in mammalian hepadnaviruses, emphasizing the importance of silencing by this complex (Abdul et al., 2018). In keeping with its essential housekeeping function, the SMC5/6 complex shows only weak overall signatures of positive selection (Abdul et al., 2018), similar to the SERINC family of restriction factors (Murrell et al., 2016) but different from the canonical arms race dynamics seen for more classical restriction factors.

The antagonism of SMC5/6 by both HBV, and primate lentiviruses, provides an unusual example of convergent evolution in two unrelated nuclear viruses. Other DNA viruses, including herpesviruses, also need to overcome the host-mediated epigenetic silencing of their extrachromosomal, episomal DNA. It is perhaps not surprising that the mammalian nucleus presents such a hostile environment to invading pathogens, as foreign DNA provides a very real threat to both cellular and genome integrity. The chromatinization and epigenetic silencing of extrachromosomal DNA therefore provides a critical first line defense against nuclear invasion. To counteract this host defense, the acquisition of accessory genes equips complex primate lentiviruses with the necessary tools to modulate the host cell environment and escape cellular restriction. Despite their ability to antagonize these extrachromosomal silencing pathways, lentiviral integration into the host genome provides an alternative route to escape nuclear immunosurveillance and extrachromosomal restriction, which may have contributed to the extraordinary success of these viruses.

Our study provides the first description of a silencing pathway that specifically targets unintegrated HIV-1 genomes. The Goff lab recently linked silencing of unintegrated MLV retroviral genomes to NP220-dependent recruitment of the HUSH complex (Zhu et al., 2018), an epigenetic silencing complex we previously showed can repress integrated lentivirus expression (Tchasovnikarova et al., 2015). However, in concordance with the Goff lab observations (Zhu et al., 2018), we did not find a role for the HUSH complex in silencing unintegrated HIV-1. Instead, our screen identified a critical role for SLF2 and the SMC5/6 complex in the silencing of unintegrated lentiviral genomes, which is both HUSH and NP220 independent.

SMC family proteins are ATP-dependent molecular motors that play central roles in regulating chromatin structure and genome stability. The SMC5/6 complex is best recognized for its role in DNA repair but is also essential for the maintenance of cellular DNA repeat regions (Peng et al., 2018; Torres-Rosell et al., 2005). In *S. cerevisiae*, the SMC5/6 complex functions in chromatin silencing of non-coding telomeric and ribosomal DNA repeat sequences, independent of its role in homologous recombination, and depletion of SMC5/6 leads to silencing defects and reduced repeat stability (Moradi-Fard et al., 2016, 2020). The mechanism of SMC5/6 complex recruitment is poorly understood. In *S. pombe*, the SLF2 ortholog Nse6 is a DNA-loading factor for SMC5/6 and plays a key role in the recruitment and chromatin loading of the SMC5/6 complex (Etheridge et al., 2020; Oravcová et al., 2018). This is consistent with our finding that SLF2 recruits the SMC5/6 complex to unintegrated HIV-1 genomes and that depletion of SLF2, and each individual component of the SMC5/6 complex, enhances unintegrated viral gene expression. In contrast, viral gene expression was unaffected by the loss of either SLF1 or the RAD18-RNF8-RNF168 ubiquitin signaling axis, factors that recruit the SMC5/6 complex to sites of DNA damage (Räschle et al., 2015). We therefore postulate that SLF2 recruits the SMC5/6 complex to extrachromosomal viral genomes for chromatin compaction, a function that is independent of its role in the DNA repair pathway.

Cohesin and condensin, two of the better-characterized members of the SMC family, compact nucleosome-bound DNA by loop extrusion (Kim et al., 2019; Terakawa et al., 2017) and led us to propose that the SMC5/6 complex uses a similar chromatin compaction-based mechanism to silence unintegrated viral DNA. Our ATAC-seq experiments showed that Vpr-mediated SLF2 depletion rendered unintegrated viral chromatin more accessible to the Tn5 transposase, revealing reduced chromatin compaction. Vpr therefore releases unintegrated viral DNA from the chromatin compaction imposed by the SMC5/6 complex. The 1.9-fold increase in ATAC-seq read counts upon SLF2 depletion is of similar magnitude to changes reported on chromatin accessibility following depletion of chromatin remodelers, such as the SWI/SNF complex component ARID1A (Liu et al., 2020). These changes are therefore consistent with a functionally relevant effect on silencing. Decompaction of otherwise inaccessible unintegrated viral chromatin will allow access of additional chromatin remodeling enzymes, leading to increased activating histone marks (H3K4me3 and H3K9ac) and gene expression. The low level of H3K9me3 we detected may reflect an independent layer of silencing and was neither HUSH dependent nor affected by SLF2 depletion. More direct evidence for a role for SMC5/6 in DNA compaction comes from two very recently published *in vitro* studies. Single-molecule magnetic tweezer assays showed that both yeast (Gutierrez-Escribano et al., 2020) and human (Serrano et al., 2020) SMC5/6 complexes were able to compact DNA in an ATP-dependent fashion. These biophysical studies therefore support a functional role for the SMC5/6 complex in compacting chromatinized, extrachromosomal viral DNA.

Exactly how SLF2 recruits the SMC5/6 complex to extrachromosomal viral DNA remains unclear. The recently published *in vitro* DNA compaction studies showed preferential SMC5/6 binding to and compaction of DNA with unusual tertiary structures such as supercoiling (Gutierrez-Escribano et al., 2020; Serrano et al., 2020). These DNA structures may form the basis for SMC5/6 recognition of extrachromosomal viral DNA, especially given the known supercoiling of HBV cccDNA, which is similarly restricted by the SMC5/6 complex (Decorsière et al., 2016; Murphy et al., 2016). A sequence-independent recruitment mechanism could allow the SMC5/6 complex to silence different forms of viral as well as cellular extrachromosomal DNA (ecDNA). Aberrant recombination of repetitive DNA regions is a major source of cellular ecDNA (Cohen and Segal, 2009). The SMC5/6 complex limits recombination at these regions (Torres-Rosell et al., 2005) and, in the light of our results, may also act to compact and silence any ecDNA released as a result of inappropriate recombination. Given the high prevalence of additional viral and cellular extrachromosomal DNAs, we predict that the impact of this silencing mechanism is likely to extend beyond the confines of HIV biology.

## STAR★Methods

### Key resources table

**Table:** 

REAGENT or RESOURCE	SOURCE	IDENTIFIER
**Antibodies**		

Rabbit anti-SLF2	Abcam	Cat#ab122480; RRID: AB_11129755
Rabbit anti-VPRBP/DCAF1	Abcam	Cat#ab202587; RRID: RRID: AB_2885060
Rabbit anti-Histone H3	Abcam	Cat#ab1791; RRID: AB_302613
Rabbit anti-Histone H3K9me3	Abcam	Cat#ab8898; RRID: AB_306848
Rabbit anti-HLTF	Bethyl Laboratories	Cat#A300-230A; RRID: AB_2117307
Mouse anti-CD4-APC	Biolegend	Cat#317416; RRID: AB_571945
Mouse anti-LNGFR-PE	Biolegend	Cat#345106; RRID: AB_2152647
Rabbit anti-Histone H3K4me3	Cell Signaling	Cat#9751; RRID: AB_2616028
Rabbit anti-Histone H3K9ac	Cell Signaling	Cat#9649; RRID: AB_823528
Rabbit anti-Histone H3K27me3	Cell Signaling	Cat#9733; RRID: AB_2616029
Rabbit anti-SMC6	GeneTex	Cat#GTX116832; RRID: AB_10630494
Rabbit anti-NSMCE1	GeneTex	Cat#GTX107136; RRID: AB_1951030
Mouse anti-Vif	NIH AIDS Reagent Program	Cat#6459; (Simon et al., 1995)
Mouse anti-UNG2	Origene	Cat#TA503563; RRID: AB_11126624
Rat anti-HA	Roche	Cat#11867423001; RRID: AB_390918
Goat anti-lamin B1	Santa Cruz	Cat#sc-6217; RRID: AB_648158
Mouse anti-β-actin	Sigma-Aldrich	Cat#A5316; RRID: AB_476743
Rabbit anti-ANKRD32/SLF1	Sigma-Aldrich	Cat#SAB2701555; RRID: AB_2885061
Goat anti-mouse HRP	Jackson ImmunoResearch	Cat#115-035-146; RRID: AB_2307392
Goat anti-rabbit HRP	Jackson ImmunoResearch	Cat#111-035-144; RRID: AB_2307391
Goat anti-rat HRP	Jackson ImmunoResearch	Cat#112-035-143; RRID: AB_2338138

**Bacterial and Virus Strains**		

pLTR-Tat-IRES-GFP	Eric Verdin	pEV731
pHRSIN.pSFFV-GFP	This paper	N/A
pHRSIN.pSFFV-mCherry	This paper	N/A
pHRSIN.pSFFV-iRFP	This paper	N/A
pNL4-3-ΔEnv-Nef-P2A-SBP- ΔLNGFR (NL4-3^LNGFR^)	(Naamati et al., 2019	N/A
pNL4-3-ΔEnv-Nef-P2A-SBP- ΔLNGFR-ΔVpr (ΔVpr NL4-3^LNGFR^)	(Naamati et al., 2019)	N/A
pNL4-3-ΔEnv-eGFP (NL4-3^GFP^)	NIH AIDS Reagent Program, Drs Haili Zhang, Yan Zhou, and Robert Siliciano (Zhang et al., 2004)	Cat#11100
pNL4-3-ΔEnv-eGFP-ΔVpr (ΔVpr NL4-3^GFP^)	(Greenwood et al., 2019)	N/A

**Chemicals, Peptides, and Recombinant Proteins**		

Raltegravir	Cayman Chemical	Cat#16071
MLN4924	Millipore	Cat#5054770001
IL-2	PeproTech	Cat#200–02
7-AAD	Stratech	Cat#17501
Protein G magnetic beads	Pierce	Cat#88848
Anti-HA magnetic beads	Pierce	Cat#88837
SYBR Green PCR master mix	Applied Biosystems	Cat#4309155
AMPure XP	Beckman Coulter	Cat#A63881

**Critical Commercial Assays**		

RNAscope ISH reagent kit	ACD	
Dynabeads Untouched Human CD4 T Cells kit	Invitrogen	Cat#11346D
Dynabeads Human T-Activator CD3/CD28	Gibco	Cat#11132D
Dynabeads Biotin Binder	Invitrogen	Cat#11047
TDE1 Tagment DNA Enzyme	Illumina	Cat#20034197

**Deposited Data**		

CRISPR-Cas9 KO screen data	This paper	GEO: GSE156630
ATAC-seq data	This paper	GEO: GSE156630

**Experimental Models: Cell Lines**		

CEM-T4	NIH AIDS Reagent Program, Dr JP Jacobs (Foley et al., 1965)	Cat. #117
Jurkat T cells	ATCC	Clone E6-1, TIB-152
HEK293	Lehner Lab stock	RRID: CVCL_0063
Oligonucleotides		
iRFP ChIP, forward primer: 5’- CTTCGATCGGGTGATGATCT	This paper, Sigma-Aldrich	N/A
iRFP ChIP, reverse primer: 5’- GCAGGCCTAGTTTTGACTCG	This paper, Sigma-Aldrich	N/A

**Recombinant DNA**		

Vpr target sgRNA library, see sgRNA sequences in Table S1	This paper	N/A
pKLV-U6-sgRNA.pGK-Puro-2A-BFP; see sgRNA sequences in Table S2	This paper	N/A
pCMV.SPORT6-mCherry	This paper	N/A
pCMV.SPORT6-Vpr	This paper	N/A
pCMV.SPORT6-Vpr(Q65R)	This paper	N/A
pCMV.SPORT6-Vpr(H71R)	This paper	N/A
pHR-SIREN-shControl.pGK-HygroR (GTTATAGGCTCGCAAAAGG)	(Greenwood et al., 2019)	N/A
pHR-SIREN-shDCAF1.pGK-HygroR (GTTATAGGCTCGCAAAAGG)	(Greenwood et al., 2019)	N/A
pHRSIN.pSFFV-SLF2.pGK-PuroR	This paper	N/A
pHRSIN.pSFFV-SLF2(590-1173).pGK-PuroR	This paper	N/A
pHRSIN.pRSV-3xHA-Vpr.pUb-Emerald	(Greenwood et al., 2019)	N/A
pHRSIN.pRSV-HA-Vpr(NL4-3).pUb-Emerald	(Greenwood et al., 2019)	N/A
pHRSIN.pRSV-HA-Vpr(SIVcpzPtt).pUb-Emerald	(Greenwood et al., 2019)	N/A
pHRSIN.pRSV-HA-Vpr(rcm).pUb-Emerald	(Greenwood et al., 2019)	N/A
pHRSIN.pRSV-HA-Vpr(agm).pUb-Emerald	(Greenwood et al., 2019)	N/A
pHRSIN.pRSV-HA-Vpr(mus).pUb-Emerald	(Greenwood et al., 2019)	N/A
pHRSIN.pRSV-HA-Vpr(smm).pUb-Emerald	(Greenwood et al., 2019)	N/A
pHRSIN.pRSV-HA-Vpr(HIV-2 7312a).pUb-Emerald	(Greenwood et al., 2019)	N/A
pHRSIN.pSFFV-3xHA-HBx.pGK-PuroR	This paper	N/A
pHRSIN.pSFFV-3xHA-NSMCE2.pGK-PuroR	This paper	N/A

**Software and Algorithms**		

FlowJo 10.7.1	FlowJo, LLC	RRID: SCR_008520
Prism 8.4.2	Graphpad	RRID: SCR_002798
Gen5	Biotek	RRID: SCR_017317
Bowtie2	(Langmead and Salzberg, 2012)	RRID: SCR_005476
FastX Toolkit	Hannon laboratory	RRID: SCR_005534
MAGeCK	(Li et al., 2014)	https://bitbucket.org/liulab/mageck/src/master/
Proteome Discoverer 2.1	Thermo Scientific	RRID: SCR_014477
FastQC	Babraham Bioinformatics, 2010	https://www.bioinformatics.babraham.ac.uk/projects/fastqc/
cutadapt	(Martin, 2011)	RRID: SCR_011841
BWA-MEM	(Li and Durbin, 2009)	RRID: SCR_010910
sambamba	(Tarasov et al., 2015)	https://academic.oup.com/bioinformatics/article/31/12/2032/214758
SAMtools	(Li et al., 2009)	RRID: SCR_002105
BEDTools	(Quinlan and Hall, 2010)	RRID: SCR_006646
ggplot2	(Wickham, 2016)	RRID: SCR_014601
ATACseqQC	(Ou et al., 2018)	DOI:10.18129/B9.bioc.ATACseqQC
IGV 2.8.0	(Thorvaldsdóttir et al., 2013)	RRID: SCR_011793
Detailed data analysis algorithms deposited on:	This paper	https://github.com/LDUP92/hiv-compaction

### Resource availability

#### Lead contact

Further information and requests for resources and reagents should be directed to and will be fulfilled by the Lead Contact, Paul J. Lehner (pjl30@cam.ac.uk).

#### Materials availability

All unique reagents generated in this study can be obtained upon reasonable request to the Lead Contact.

#### Data and code availability

Sequencing data associated with the CRISPR-Cas9 knockout screen and ATAC-seq have been deposited in GEO (GEO: GSE156630). Details of software and code used for analysis of CRISPR-Cas9 knockout screen and ATAC-seq have been deposited in a public repository on https://github.com/LDUP92/hiv-compaction.

### Experimental model and subject details

#### Cell lines

CEM-T4 T cells (female) were acquired from the NIH AIDS Reagent Program (Cat. #117) and Jurkat T cells (male) from ATCC (Clone E6-1, TIB-152). HEK293T cells (female) were from the Lehner lab stock. All cell lines were cultured at 37°C and 5% CO_2_ in Iscove's Modified Dulbecco's Medium (IMDM, Sigma-Aldrich) supplemented with 10% fetal calf serum (FCS, Gibco), 1x GlutaMAX (Gibco), and 100 U/mL penicillin/streptomycin (Gibco). All cell lines were regularly tested and confirmed to be mycoplasma negative (MycoAlert, Lonza).

#### Primary cells

Primary human CD4^+^ T cells were isolated from peripheral blood by density gradient centrifugation over Lympholyte-H (Cedarlane Laboratories) and negative selection using the Dynabeads Untouched Human CD4 T Cells kit (Invitrogen) following the manufacturer’s instructions. Cells were activated using Dynabeads Human T-Activator CD3/CD28 beads (Gibco) and cultured in RPMI supplemented with 10% FCS, 30 U/mL recombinant human IL-2 (PeproTech), 100 U/mL penicillin/streptomycin (Gibco) at 37°C and 5% CO_2_. Ethical permission for this study was granted by the University of Cambridge Human Biology Research Ethics Committee (HBREC.2017.20). Written informed consent was obtained from all volunteers prior to providing blood samples.

### Method details

#### Lentiviral reporters and expression vectors

Fluorescent lentiviral reporters were pHRSIN.pSFFV-GFP, pHRSIN.pSFFV-mCherry, pHRSIN.pSFFV-iRFP, and pLTR-Tat-IRES-GFP (pEV731, a gift from Eric Verdin). Full-length and Vpr deletion NL4-3 reporters were pNL4-3-ΔEnv-Nef-P2A-SBP-ΔLNGFR (NL4-3^LNGFR^) for immunostaining and flow cytometry (Naamati et al., 2019), and pNL4-3-ΔEnv-eGFP (NL4-3^GFP^) for viral RNA FISH (Greenwood et al., 2019; Zhang et al., 2004). Full-length SLF2 and NSMCE2 cDNAs were isolated by PCR amplification of the respective CDS from HeLa cDNA and cloned into pHRSIN.pSFFV-GOI.pGK-PuroR with or without a 3xHA-tag. For cloning of HA-tagged minimal SLF2, the 590-1173 amino acid region of SLF2 was amplified by PCR, appending an N-terminal SV40 NLS. Human and primate lentiviral Vpr constructs were expressed from pHRSIN.pRSV-HA-GOI.pUb-Emerald as previously described (Greenwood et al., 2019). 3xHA-HBx was cloned from a subtype A HBx construct obtained from Christine Neuveut and expressed from a pHRSIN-based vector. Non-lentiviral expression constructs pCMV-SPORT6-mCherry and pCMV-SPORT6-Vpr(NL4-3) were used for expression of protein for packaging into virus-like particles (VLP) in the absence of a viral genome.

#### Virus production and infection

VSV-G pseudotyped lentivirus was produced by transfection of HEK293T cells with a lentiviral expression vector and packaging vectors pCMVΔR8.91 and pMD.G at a DNA ratio of 3:2:1 using TransIT-293 (Mirus) following the manufacturers recommendation. For NL4-3 reporters, transfections were performed using FuGENE 6 (Promega) and a pNL4-3 to pMD.G DNA ratio of 9:1. 48 hours post transfection, supernatants were collected, filtered (0.45 μm pore size), and stored at -80°C. For sensitive applications, lentiviral supernatants were DNase treated (1 h, 37°C; RQ1, Promega) and purified (Lenti-X, Takara). Control VLPs and Vpr VLPs were produced by co-transfection of pCMV-SPORT6-mCherry or -Vpr with pCMVΔR8.91 and pMD.G at a DNA ratio of 2:2:1. All infections were performed by spinoculation (750xg, 60 min, 37°C). Chemical inhibitors used were as follows: Raltegravir (RAL, Cayman Chemical; 1 μM) and MLN4924 (Millipore; 1 μM).

#### Flow cytometry and fluorescence activated cell sorting

Flow cytometry data was collected on an LSR Fortessa (BD) and was analysed using FlowJo v.10.7.1. Geometric mean fluorescence intensities (MFI) were used for quantification. For NL4-3 flow cytometry, cells were first incubated with the indicated fluorochrome-conjugated antibodies (15 min, 4°C) and fixed in PBS/1% paraformaldehyde. For FACS, cells were resuspended in 10% FCS-PBS, filtered through a sterile 50 μm cell strainer, and sorted on an Influx (BD) or FACS Melody (BD) cell sorter into complete IMDM media supplemented with 50% FCS.

#### In situ viral RNA detection and imaging

HIV-1 RNA detection was performed by branched DNA in situ hybridization (bDNA FISH) following a modified RNAscope protocol with RNAscope reagents from Advanced Cell Diagnostics (ACD) (Wang et al., 2012). Briefly, cells were seeded on poly-d-lysine coated coverslips 48 h post infection, fixed in 4% PFA (30 min, RT), washed three times in PBS, incubated 10 minutes in 0.1% Tween-20-PBS (PBS-T), and washed twice in PBS. Cells were incubated with manufacturers protease treatment (Pretreat 3; 1:5 dilution in PBS) in a humidified ACD HybEZ oven at 40°C, 15 min. Protease solution was decanted, and samples were washed twice in PBS. A probe that recognizes HIV-1 RNA (HIV-nongagpol-C3; ACD 317711-C) was applied following manufacturers recommendations and samples incubated at 40 °C for 2 h in the HybEZ oven. Remaining wash steps, hybridization of preamplifiers, amplifiers, and fluorescent label were performed as previously described (Puray-Chavez et al., 2017). Nuclei were counter-stained with 4’,6’-diamino-2-phenylinndole (DAPI) and mounted using Prolong Gold Antifade (Invitrogen). Imaging was performed using a Nikon C2 confocal microscope using a 60x APO oil-immersion objective (numerical aperture 1.4). The excitation/emission bandpass wavelengths to detect DAPI (405 nm) and HIV-1 RNA (647 nm) were set to 420-480 nm and 655-705 nm, respectively. Images were quantified using Gen5 software (BioTek) to count individual cells and determine the integrated fluorescence intensity of HIV-1 RNA per cell. Background signal was determined using uninfected Jurkat T cells processed as described above.

#### Vpr target library

To design a sub-genomic Vpr target sgRNA library, we identified 1,217 genes encoding proteins depleted by at least 30% in presence of Vpr in a number of published (Greenwood et al., 2016, 2019) and unpublished proteomics datasets. For each gene,10 sgRNA sequences were identified from published genome-wide sgRNA libraries (Morgens et al., 2017; Wang et al., 2014). Library composition and sgRNA sequences can be seen in Table S1. sgRNAs were synthesised as a pooled oligonucleotide array (CustomArray) and cloned into pKLV-U6-sgRNA(BsmBI-stuffer).pGK-Puro-2A-BFP (modified from Addgene #50946) as reported previously (Doench et al., 2016). Essentially, sgRNA pools were amplified by PCR, digested with BsmBI, cloned into pKLV using T7 DNA ligase, and amplified in Stbl4 electrocompetent cells.

#### CRISPR-Cas9 knockout screen

48 x 10^6^ Cas9-CEM-T4 cells were transduced with “Vpr target library” sgRNA lentivirus at MOI ∼ 0.3. Transduction efficiency was verified by flow cytometry (BFP^+^) 72 hpi, and sgRNA containing cells were enriched by puromycin selection. On day 7, 48 x 10^6^ cells were transduced at MOI ∼ 1.5 with pHRSIN.SFFV-GFP and pHRSIN.SFFV-mCherry by spinoculation in complete IMDM + 1 μM raltegravir (RAL) and were subsequently maintained in 1 μM RAL for the duration of the screen. The top 0.5% GFP^+^/mCherry^+^ cells were selected by fluorescence activated cell sorting (FACS) on day 10, and cells with stably integrated virus were removed by sorting the GFP^-^/mCherry^-^ population on day 16. On day 18 and 21, respectively, reporter virus infection and sorting of highly expressing cells were repeated, and DNA was isolated immediately after sorting (Zymo Quick-DNA Microprep). An uninfected, unsorted library population was maintained separately at >150-fold library coverage for the duration of the screen, and unsorted library DNA was isolated for reference (Qiagen, Gentra Puregene). sgRNA sequences were amplified and Illumina sequencing adaptors added by two sequential rounds of PCR followed by PCR purification (AMPure XP, Beckman Coulter). Next-generation sequencing was performed on a MiniSeq System (Illumina) using a custom primer. For data analysis, single-end 35 bp reads were trimmed down to the variable sgRNA segment using FASTX-Toolkit and aligned to an index of all sequences in the Vpr target library using Bowtie 2. Read count statistics were generated using the MAGeCK algorithm (Li et al., 2014). The data analysis script is available on: https://github.com/LDUP92/hiv-compaction. Sequencing data is available from GEO (GEO: GSE156630).

#### CRISPR-mediated gene knockout

CRISPR/Cas9 mediated genomic editing was performed by transduction of cell lines stably expressing Cas9 with pKLV-U6-sgRNA(BbsI)-PGK-Puro-2A-BFP vectors encoding sgRNA sequences targeting the gene of interest. sgRNA sequences are listed in Table S2. Phenotypic experiments of resulting mixed knockout populations were performed on puromycin selected cells immediately 7 days post sgRNA introduction to minimise lethality-based outgrowth effects. For generation of clonal knockout cell lines, sgRNA transduction was performed in 1 μM RAL to prevent stable integration, and single cell clones were isolated in round-bottom 96-well plates on a BD FACSMelody. For unintegrated virus reporter assays in mixed and clonal knockout populations, BFP^+^ WT Jurkat cells were mixed 1:1 with BFP^-^ knockout cells prior to reporter infection to control infection levels.

#### Validation of clonal knockout cell lines

SLF2 and SLF1 knockout clone cell lines were identified through screening of >100 clones by immunoblotting. The isolated knockout clones had normal cell morphology and only a mild decrease in cell proliferation compared to the parental WT Jurkat T cells. To confirm biallelic gene disruption, genomic DNA was isolated from 1 x 10^6^ cells using a QIAamp DNA Mini Kit (Qiagen). The sgRNA target exons were amplified by nested PCR using the following primer combinations: *SLF1*, exon 11; outer PCR: 5’- GCAGTTCCAGGAACAATTTGGA, 5’- AACACTTCGGGGCATTGATG; inner PCR: 5’-TCTTTGCTGTGGTTAACATGGT, 5’- GCCAAGACTTCAAACACATGAC. *SLF2*, exon 5; outer PCR: 5’- TGTTTGTTTTAGGGAGTGGCA, 5’-GCACAACTTCCAAAGCAGCA; inner PCR: 5’-TGGAATGAAAATGAGCATTTGTCA, 5’- TCTGTAGAATGCCCAGAACATT. PCR products were resolved by gel electrophoresis, gel purified, and prepared for Sanger sequencing using a Zero Blunt TOPO PCR cloning kit (Invitrogen). 10 colonies were sequenced for each knockout clone to cover all alleles.

#### Immunoblotting

Cells were lysed in Laemmli buffer supplemented with 100 mM dithiothreitol (DTT) and benzonase (1:100, Sigma-Aldrich). Following denaturation at 65°C, samples were separated by SDS-PAGE and transferred to PVDF-membranes (Millipore) by TransBlot semi-dry transfer. Membranes were blocked in 5% (w/v) milk – PBS-T (0.2% Tween-20) and incubated with primary antibody in milk-PBS-T at 4°C overnight followed by incubation with HRP-conjugated secondary antibodies at room temperature (RT). Blots were developed using chemiluminescent substrates (Thermo Scientific) and visualised using either X-ray film or an iBright CL1000 imaging system (Thermo Fisher Scientific). The following antibodies were used for immunoblotting, listed by manufacturer: Primary antibodies: Abcam: Rabbit α-SLF2 (ab122480), rabbit α-VPRBP/DCAF1 (ab202587). Bethyl Laboratories: Rabbit α-HLTF (A300-230A). GeneTex: Rabbit α-SMC6 (GTX116832), rabbit α-NSMCE1 (GTX107136). NIH AIDS Reagent Program: Mouse α-Vif (#6459; (Simon et al., 1995)). Origene: Mouse α-UNG2 (TA503563). Roche: Rat α-HA (11867423001). Santa Cruz: Goat α-Lamin B1 (sc-6217). Sigma-Aldrich: Mouse α-β-actin (A5316), rabbit α-ANKRD32/SLF1 (SAB2701555). Secondary antibodies: Jackson ImmunoResearch: Goat α-mouse-HRP (115-035-146), goat α-rabbit-HRP (115-035-144), and goat α-rat-HRP (115-035-143).

#### Immunoprecipitation and mass spectrometry

Nuclei were extracted by incubation of 15 x 10^6^ cells in cell lysis buffer (0.1% IGEPAL, 85 mM KCl, 10 mM HEPES). Isolated nuclei were pelleted and lysed in nuclei lysis buffer (1% IGEPAL, 1x TBS plus 1:100 benzonase (Sigma-Aldrich) and protease inhibitor cocktail). Lysates were clarified by centrifugation at 10,000xg, 10 min, 4°C and pre-cleared by incubation with protein A and immunoglobulin G (IgG)-sepharose (GE Healthcare, cat#17096901). For endogenous SLF2 IP, incubation with 1 μg primary antibody was performed in IP buffer (0.5% IGEPAL, 1xTBS) for 3 h at 4°C followed by 1 h incubation with protein A-sepharose, 4°C. For HA IP, lysates were incubated with 30 μL anti-HA agarose beads (Sigma-Aldrich, Cat#E6779). Following 5 washes in IP buffer, bound proteins were eluted in 2% SDS, 50 mM Tris (pH 8) at 65°C. Eluted samples were brought to 5% SDS and reduced/alkylated with 10mM TCEP/40mM Iodoacetamide. Subsequently, samples were digested on a micro S-trap (Protifi), acidified with phosphoric acid, and precipitated with neutral buffered methanol (wash buffer) before loading onto an S-trap using a vacuum manifold. Traps were washed with wash buffer prior to digest with trypsin/lysC in HEPES pH8 (digestion buffer) (37°C, 6 h). Peptides were eluted sequentially with digestion buffer, 0.2% formic acid and 0.2% formic acid + 50% Acetonitrile. Eluted samples were dried in a vacuum centrifuge and stored at -20°C prior to analysis. Mass spectrometry data acquisition and analysis were performed as previously described (Greenwood et al., 2019). Briefly, data were acquired on an Orbitrap Fusion (Thermo Fisher) operating a 1 h reversed phase gradient. The instrument obtained MS1 spectra in the Orbitrap and MS2 spectra in the ion trap. Data were searched using Mascot (Matrix Science) from within Proteome Discoverer (Thermo Fisher), and Percolator was used to determine PSM FDR which was controlled at 1%. Proteins were quantified using the Hi3 method.

#### 7-AAD staining

Cells were washed in PBS and fixed in ice cold 70% ethanol, 30 min, followed by 2x washes with 1% BSA-PBS. Fixed cells were stained in 25 μg/mL 7-AAD on ice for 30 min followed by flow cytometry analysis.

#### Chromatin immunoprecipitation

10 x 10^6^ cell aliquots were crosslinked in 1% formaldehyde in complete IMDM (10 min, RT) and quenched with 0.125 M glycine (5 min, RT). Nuclei were isolated by lysis in 2x 5 mL ChIP lysis buffer (10 mM HEPES, 85 mM KCl, 0.5% IGEPAL, protease inhibitor cocktail) at 4°C, 5 min, and nuclei pelleted (1,000xg, 5 min, 4°C). Nuclei were lysed in 200 μL MNase buffer (10 mM Tris-HCl pH 7.5, 10 mM NaCl, 3 mM MgCl_2_, 1 mM CaCl_2_, 4% IGEPAL, protease inhibitor cocktail) supplemented with 1 μL MNase and 1 μg RNase A, incubated 10 min at 37°C, and the digest immediately quenched by addition of 100 μL MNase-STOP buffer (50 mM Tris-HCl pH 8.1, 10 mM EDTA, 10 mM EGTA, 1% SDS, protease inhibitor cocktail) 5 min, 4°C. Digested chromatin was sonicated in 1.5 mL tubes in a Bioruptor Pico (Diagenode) for 5 cycles (10 s ON, 30 s OFF, 4°C) and clarified by centrifugation at 6,000xg, 10 min, 4°C. Lysates were diluted by adding 1 mL IP dilution buffer (20 mM Tris-HCl, 2 mM EDTA, 150 mM NaCl, 1% Triton X-100, 0.01% SDS, protease inhibitor cocktail) and pre-cleared by incubation with 12.5 μL Protein G magnetic beads (Pierce), 4°C, 2 h. 120 μL pre-cleared chromatin was kept as input, equivalent to 1 x 10^6^ cells. Chromatin aliquots from 5 x 10^6^ cells for histone ChIP or 10 x 10^6^ cells for HA-NSMCE2 ChIP were immunoprecipitated with 5 μg primary antibody and 12.5 μL Protein G magnetic beads or 30 uL magnetic anti-HA beads (Pierce), 4°C, overnight. Bead-bound chromatin was washed twice in low-salt buffer (20 mM Tris-HCl pH 8.1, 2 mM EDTA, 50 mM NaCl, 1% Triton X-100, 0.1% SDS), once in LiCl buffer (10 mM Tris-HCl pH 8.1, 1 mM EDTA, 250 mM LiCl, 1% IGEPAL, 1% sodium deoxycholate monohydrate), and twice in TE buffer (10 mM Tris-HCl pH 8.1, 1 mM EDTA). Protein-DNA complexes were eluted from beads in 200 μL elution buffer (1% SDS, 100 mM NaHCO_3_) and de-crosslinked with 0.3 M NaCl (final) and 1 μg RNase A (65°C, overnight), followed by digestion with 3 μL proteinase K and PCR purification (Qiagen). qPCR was performed with SYBR green mastermix (Applied Biosystems) and iRFP primers:

5’- CTTCGATCGGGTGATGATCT, 5’- GCAGGCCTAGTTTTGACTCG on a QuantStudio7 Flex (Applied Biosystems). The following antibodies were used for ChIP, listed by manufacturer: Abcam: Rabbit α-Histone H3 (ab1791), rabbit α-H3K9me3 (ab8898). Cell Signaling: Rabbit α-H3K4me3 (#9751), rabbit α-H3K9ac (#9649), rabbit α-H3K27me3 (#9733). Magnetic beads were purchased from Pierce: α-HA magnetic beads (#88837), Protein G magnetic beads (#88848).

#### ATAC-seq

Chromatin accessibility was assessed by ATAC-seq following the protocol detailed by Buenrostro and colleagues (Buenrostro et al., 2015), without prior nuclei isolation (Karabacak Calviello et al., 2019). Briefly, 50,000 Jurkat T cells were resuspended in 50 μL 1x Illumina Tagment DNA with 2.5 μL TDE1 Tagment DNA Enzyme (Illumina). Transposition reactions were incubated in a thermomixer (1 h, 37°C, 1,400 rpm) and chromatin fragments purified using a MinElute PCR purification kit (Qiagen). Libraries of transposed chromatin fragments were prepared by minimal PCR amplification (8 or 9 total PCR cycles) using custom indexed primers, dual size selected with AMPure XP beads (Beckman Coulter) and sequenced on a NovaSeq6000 (paired-end 150 bp reads). Sequencing reads were quality checked and trimmed using FastQC (Babraham Bioinformatics, 2010) and cutadapt (Martin, 2011). The 3.8 kB viral genome (1-LTR circle) was concatenated to the human hg38 reference genome. Reads were aligned to the combined genome using BWA-MEM (Li and Durbin, 2009) and the resulting alignments processed using sambamba (Tarasov et al., 2015) followed by filtering with SAMtools (Li et al., 2009). Reads aligning to chrM or to the ENCODE blacklists were excluded from subsequent analyses. To allow for differential analyses of normalised read count densities for both the viral and cellular genomes, 100,000 different virus-sized regions were randomly sampled using BEDTools (Quinlan and Hall, 2010) from the parts of the human genome that are mappable by ATAC-seq in an uninfected control library. Statistical testing was performed using Fisher’s tests in the R programming language. Data visualisation was performed using ggplot2 (Wickham, 2016), ATACseqQC (Ou et al., 2018) and the Integrative Genomics Viewer (Thorvaldsdóttir et al., 2013). For more details about the bioinformatics data analysis, see: https://github.com/LDUP92/hiv-compaction. Sequencing data are available on GEO (GEO: GSE156630).

### Quantification and statistical analysis

Unless otherwise stated, statistical significance was calculated using ordinary one-way ANOVA with multiple comparisons correction performed in GraphPad Prism v8. Error bars denote standard deviation. ns, *P* > 0.05. ^∗^, *P* < 0.05. ^∗∗^, *P* < 0.01. ^∗∗∗^, *P* < 0.001. Statistical parameters for each experiment are provided in figure legends. Details of statistical analysis for CRISPR-Cas9 knockout screen and ATAC-seq are provided in the respective sections and in our code repository: https://github.com/LDUP92/hiv-compaction.

## Supplemental information

Document S1. Figures S1–S7

Table S1. Vpr target sgRNA library composition, related to Figure 2

Table S2. sgRNA sequences used for individual gene knockout, related to Figures 2 and 3

Document S2. Article plus supplemental information
