# Supplementary material for: The SMC5/6 complex compacts and silences unintegrated HIV-1 DNA and is antagonized by Vpr
Source: Cell Host Microbe. Author manuscript; Available in PMC 2021 Jun 19. (PMC8118623; doi:10.1016/j.chom.2021.03.001)
Supplement: Figures S1-S7 [file NIHMS1701024-supplement-Figures_S1-S7.pdf]

Cell Host & Microbe, Volume *29*

Supplemental information

The SMC5/6 complex compacts and silences unintegrated HIV-1 DNA and is antagonized by Vpr

Liane Dupont, Stuart Bloor, James C. Williamson, Sergio Martínez Cuesta, Raven Shah, Ana Teixeira-Silva, Adi Naamati, Edward J.D. Greenwood, Stefan G. Sarafianos, Nicholas J. Matheson, and Paul J. Lehner

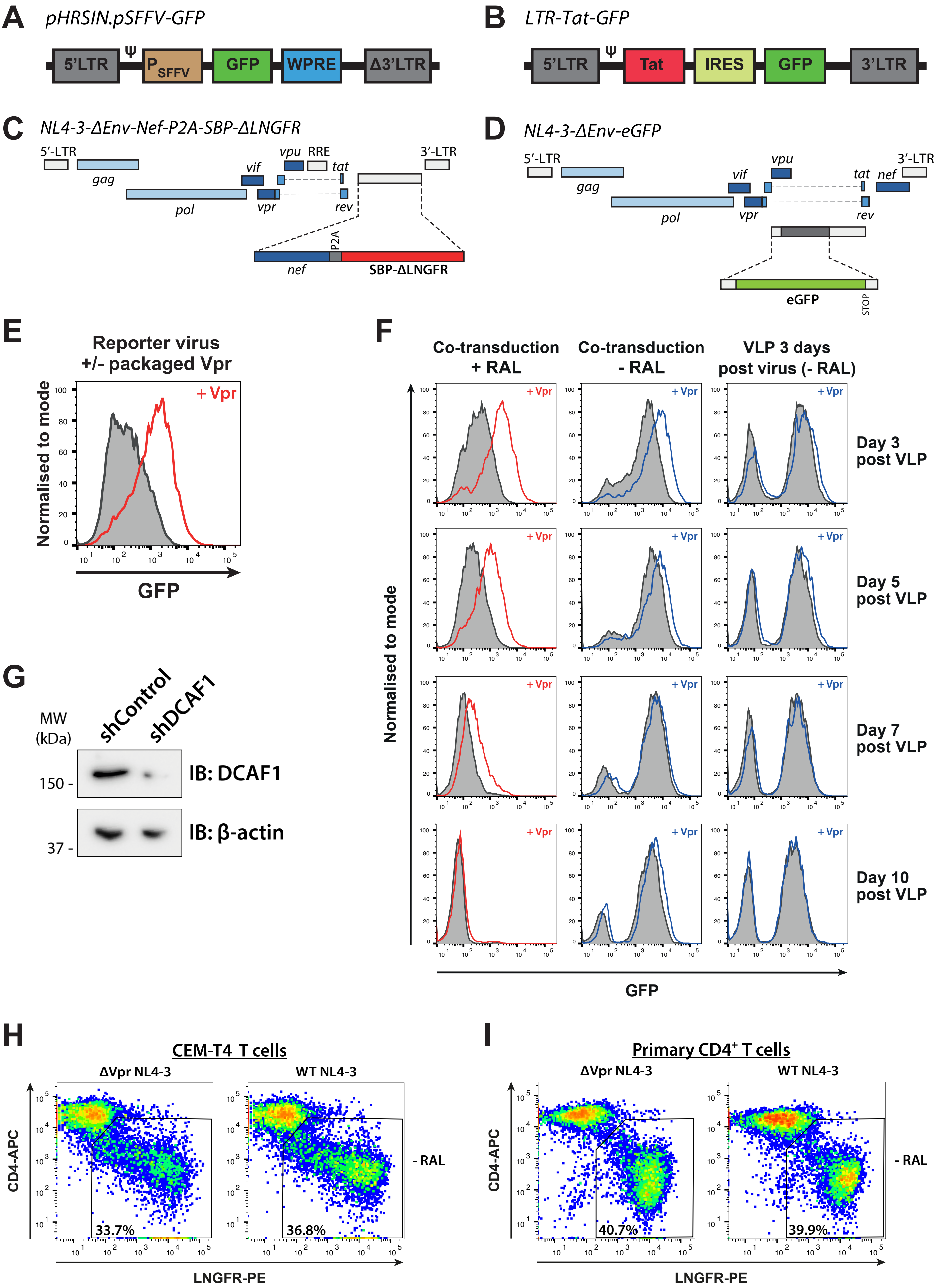

Figure S1. HIV-1 Vpr delivered in VLPs or within reporter virions increases gene expression from unintegrated but not stably integrated lentiviral reporters — Related to Figure 1

Figure S1. HIV-1 Vpr delivered in VLPs or within reporter virions increases gene expression from unintegrated but not stably integrated lentiviral reporters — Related to Figure 1 (continued)

(**A-D**) Maps of reporter viruses. (**A**) SFFV-GFP lentiviral reporter. GFP expressed from an SFFV promoter in a lentiviral expression construct (VSV-G pseudotyped). (**B**) LTR-Tat-GFP reporter. GFP expressed from a wild type HIV-1 LTR in a lentiviral reporter construct also expressing Tat (VSV-G pseudotyped). (**C**) NL4-3^{LNGFR} reporter. Full-length NL4-3 reporter with an env deletion and an LNGFR epitope inserted downstream of the nef gene, separated by a P2A self-cleaving peptide (VSV-G pseudotyped). (**D**) NL4-3^{GFP} reporter. NL4-3 reporter with env deletion and a GFP reporter inserted (VSV-G pseudotyped). (**E**) Vpr packaged in virion increases unintegrated virus gene expression. CEM-T4 T cells were infected with SFFV-GFP lentiviral reporters with or without packaged Vpr in the presence of RAL. Flow cytometry 72 hpi. Representative example (n=2). (**F**) Vpr VLPs do not increase gene expression of stably integrated lentiviral reporters. CEM-T4 T cells were transduced with SFFV-GFP lentiviral reporters either 3 days prior to or upon co-transduction with control or Vpr VLPs ± RAL and analysed by flow cytometry (n=1). (**G**) Immunoblot of DCAF1 knockdown. (**H-I**) Vpr deletion from NL4-3 reporters has only a marginal effect on viral gene expression and CD4 downregulation when integration is not inhibited. CEM-T4 T cells (**H**) or primary CD4+ T cells (**I**) were infected with WT or ΔVpr NL4-3^{LNGFR} lentiviral reporters in the absence of RAL. Cells were stained with anti-LNGFR and anti-CD4 antibodies 48 hpi and analysed by flow cytometry (n=2).

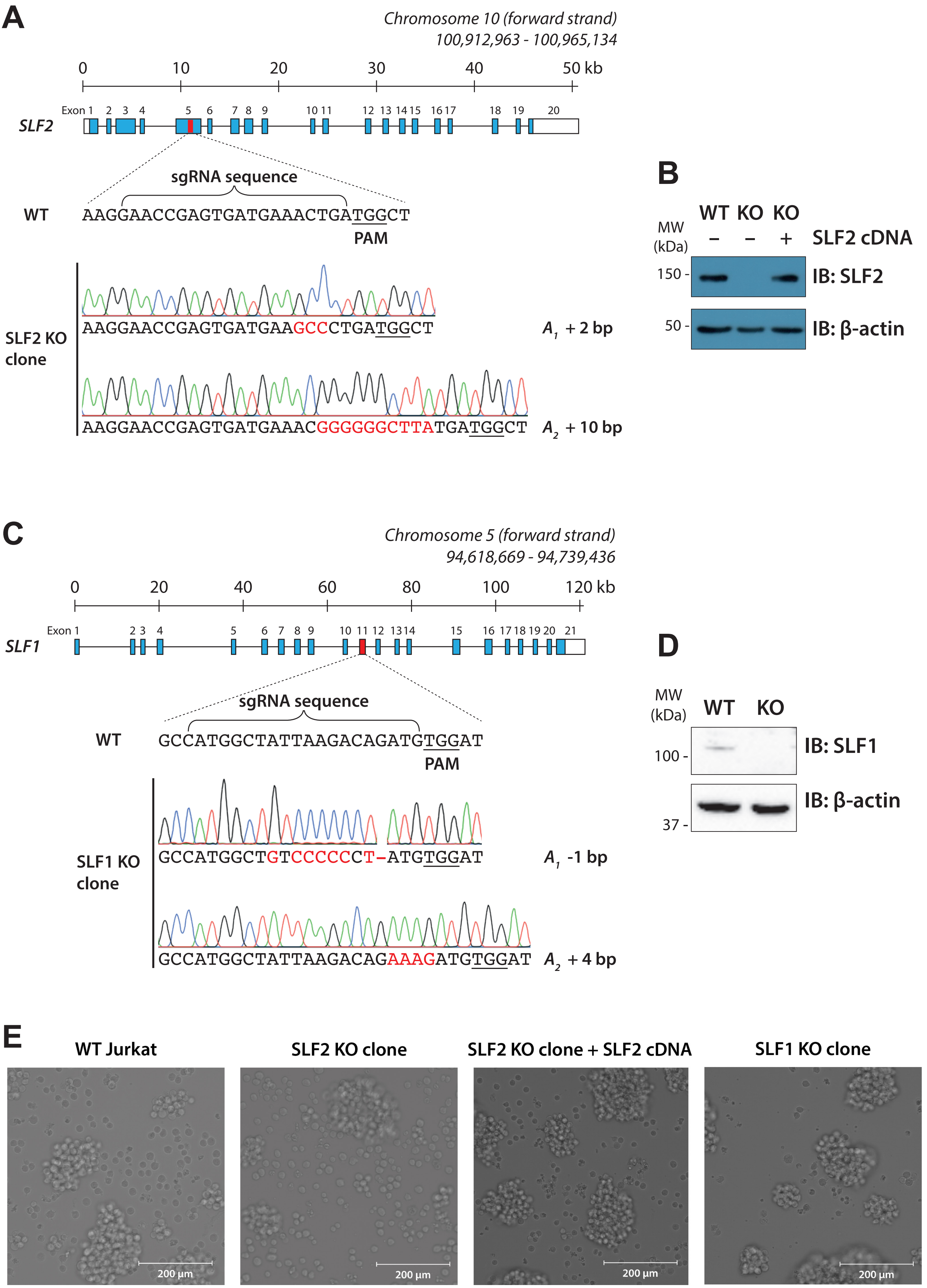

Figure S2. Validation of SLF2 and SLF1 knockout clones by Sanger sequencing and immunoblotting — Related to Figure 2 and Figure 3

Figure S2. Validation of SLF2 and SLF1 knockout clones by Sanger sequencing and immunoblotting — Related to Figure 2 and Figure 3 (continued)

(**A-B**) Validation of SLF2 KO clone. (**A**) Sanger sequencing of SLF2 sgRNA target exon. Organisation of the human SLF2 gene shown with the sgRNA target sequence highlighted. To confirm biallelic gene disruption in the isolated clonal SLF2 knockout cell line, the target exon was amplified from isolated genomic DNA, TOPO cloned, and analysed by Sanger sequencing. Sequencing tracks representative of the two alleles identified among 10 sequenced TOPO clones are shown with frameshift inducing indels highlighted. (**B**) Immunoblot of lysates from WT Jurkat, clonal SLF2 knockout cells, and clonal SLF2 knockout cells complemented with full-length SLF2 cDNA. (**C-D**) Validation of SLF1 KO clone. (**C**) Sanger sequencing of SLF1 target exon performed as described above. (**D**) Immunoblot of WT Jurkat and clonal SLF1 KO cells. (**E**) Cell morphology. Light microscopy images of WT Jurkat, SLF2 KO clone, and SLF2 cDNA complemented SLF2 KO clone cells, and SLF1 KO clone cells.

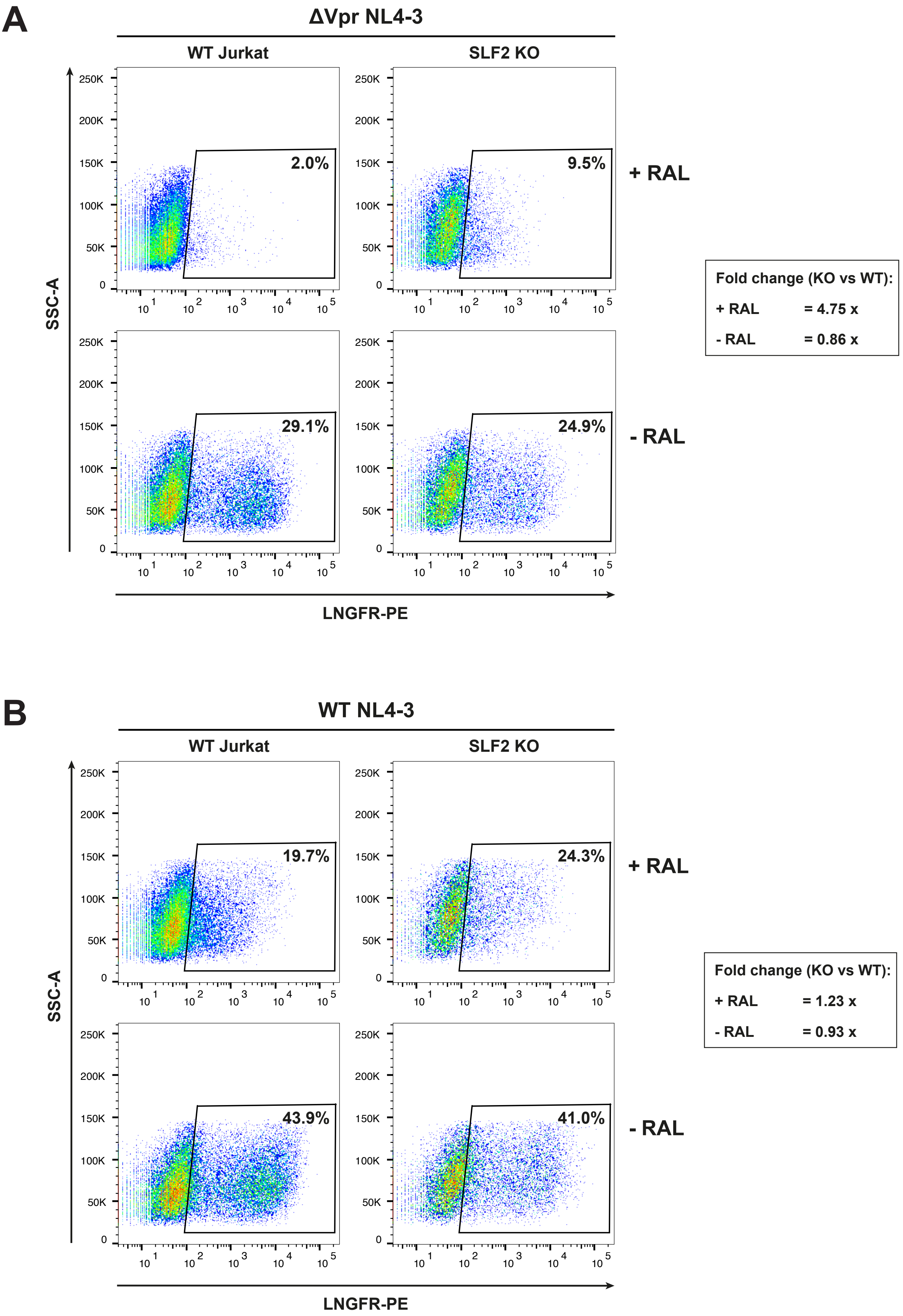

Figure S3. SLF2 knockout rescues gene expression from unintegrated Vpr-deletion NL4-3 reporter viruses — Related to Figure 2

(**A-B**) Gene expression from unintegrated Vpr deletion NL4-3 reporters is increased upon SLF2 knockout. BFP^+ WT Jurkat cells and BFP^- SLF2 knockout Jurkat cells were mixed 1:1 and infected at low MOI with ΔVpr NL4-3^{LNGFR} (**A**) or WT NL4-3^{LNGFR} (**B**) lentiviral reporters at equal MOI with or without RAL. Cells were stained with anti-LNGFR antibodies 48 h post infection and analysed by flow cytometry (n=1).

A **HHPred homology search for SLF2_human (Q8IX21)**

Query proteome: *Schizosaccharomyces pombe*

Hit ID	Hit name	Hit length	Aligned cols	Identities	E-value	Probability
NP_594933.1	Nse6	522 aa	454	13%	6.8e-36	100%

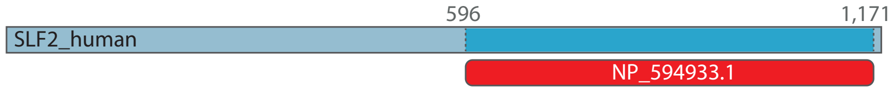

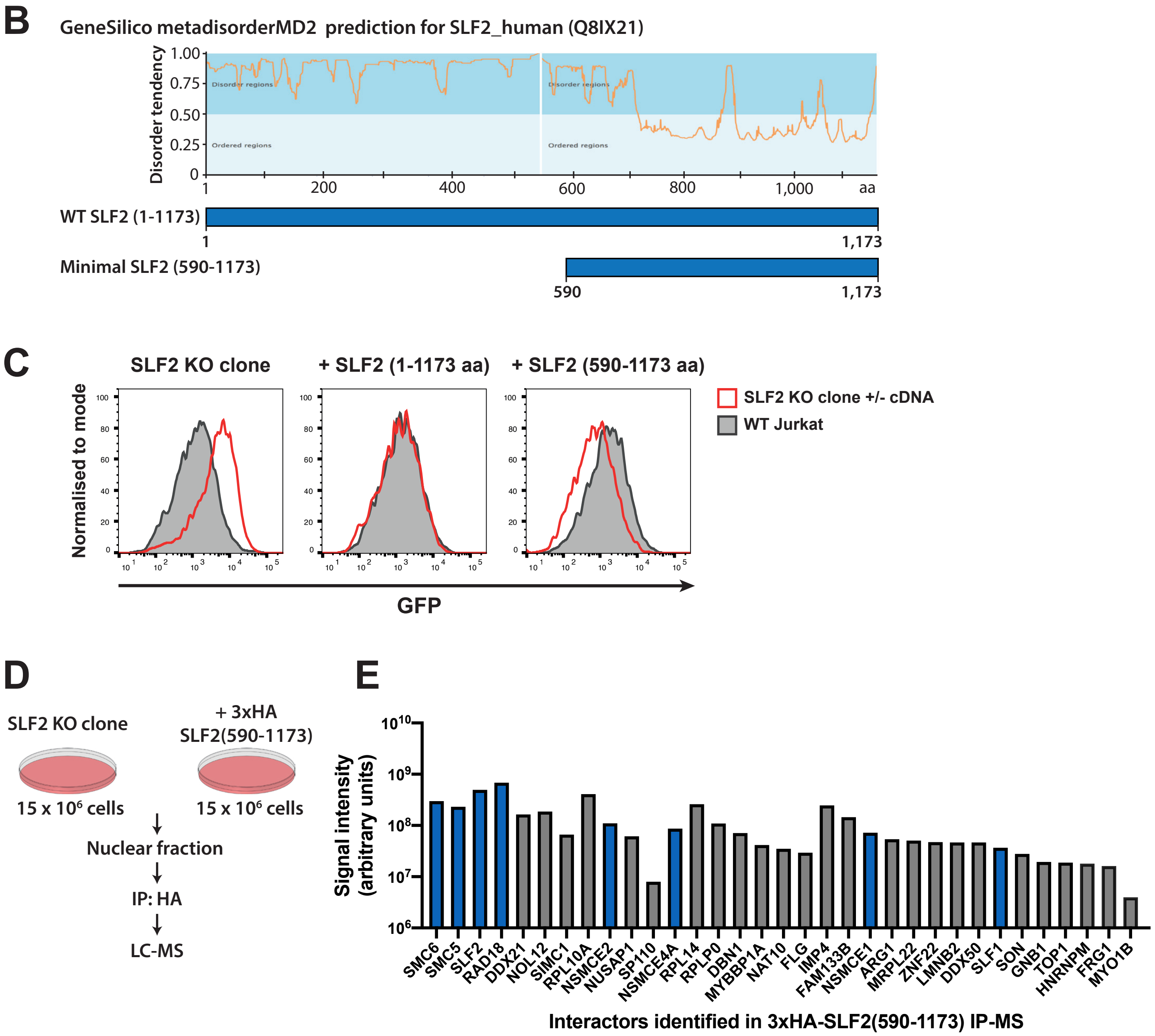

Figure S4. A truncated minimal SLF2 fully restores unintegrated virus silencing in an SLF2 knockout clone and co-immunoprecipitates with the SMC5/6 complex — Related to Figures 2 and 3

(**A**) Nse6 is the S. pombe homolog of human SLF2. A HHPred homology search was performed with human SLF2 (Q8IX21) against the S. pombe proteome using default parameters. Summarising hits with >50% confidence. (**B**) The SLF2 N-terminal is predicted to be disordered. Human SLF2 protein sequence (Q8IX21) was analysed using the GeneSilico MetadisorderMD2. Plot shows predicted disorder tendency for each position (disordered regions have scores >0.5). A minimal SLF2 was cloned by truncation based on the Nse6 homology and disorder predictions as illustrated. (**C**) Minimal SLF2 cDNA fully restores silencing in an SLF2 knockout clone. Unintegrated reporter virus assay in SLF2 knockout cells or knockout cells complemented with either full-length SLF2(1-1173 aa) or minimal SLF2(590-1173 aa). Analysis by flow cytometry 72 hpi. Data representative example of n=3. (cont.)

Figure S4. A truncated minimal SLF2 fully restores unintegrated virus silencing in an SLF2 knockout clone and co-immunoprecipitates with the SMC5/6 complex — Related to Figures 2 and 3 (continued)

(cont.) (**D-E**) IP-MS identifies SMC5/6 complex components as interactors of minimal SLF2. (**D**) Material from immunoprecipitation with a HA antibody in SLF2 knockout cells and SLF2 knockout cells complemented with 3xHA-SLF2(590-1173) was analysed by mass spectrometry. Interactors displayed in (**E**) satisfy inclusion criteria of (i) being undetected in IP from SLF2 KO cells and (ii) detected with ≥3 peptides in IP from 3xHA-SLF2(590-1173) complemented cells, ordered in descending order by number of unique peptides identified (n=1). Blue bars represent SLF2 and SMC6 interactors previously identified by (Räschle et al., 2016).

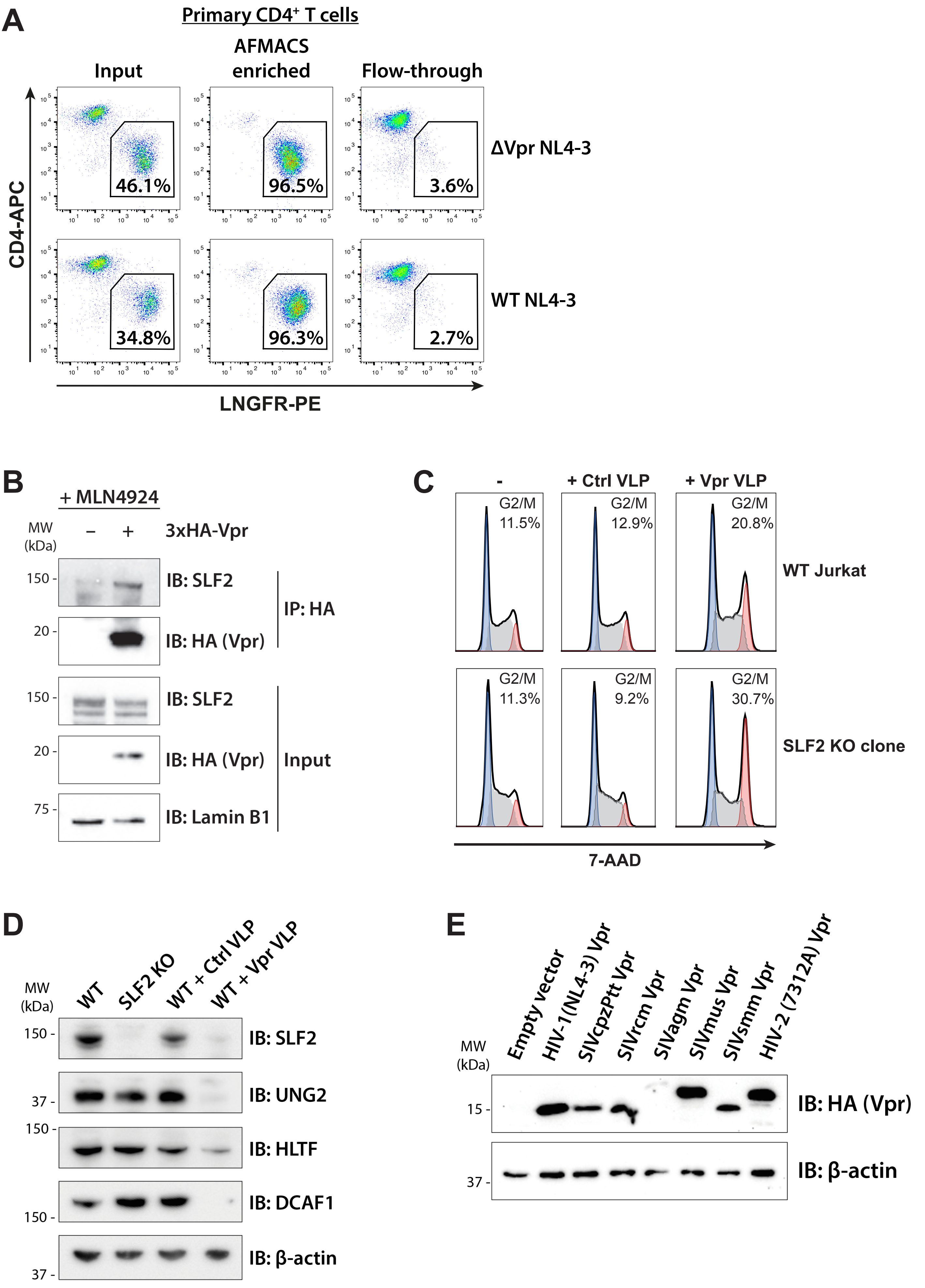

Figure S5. SLF2 is a novel Vpr target unrelated to previously characterised functions of Vpr — Related to Figure 4

Figure S5. SLF2 is a novel Vpr target unrelated to previously characterised functions of Vpr — Related to Figure 4 (continued)

(**A**) AFMACS purification of NL4-3^{LNGFR} infected primary CD4^{+} T cells. Primary CD4^{+} T cells were infected with WT or ΔVpr NL4-3^{LNGFR}. 48 hpi, LNGFR+ infected cells were enriched by antibody-free magnetic cell sorting (AFMACS). To confirm purity, unselected (input), AFMACS enriched, and flow-through cell populations were stained with α-LNGFR and α-CD4 antibodies and analysed by flow cytometry. (**B**) 3xHA-Vpr interacts with endogenous SLF2. CEM-T4 T cells were preincubated with 1 μM MLN4924 and transduced with 3xHA-Vpr. 24 h post transduction, nuclear extracts were immunoprecipitated with HA antibody conjugated magnetic beads and analysed by immunoblotting. Representative immunoblot (n=2). (**C**) Knockout of SLF2 does not affect Vpr-induced G2/M cell cycle arrest. WT Jurkat T cells and clonal SLF2 KO cells were transduced with control or Vpr VLPs. 24 hpi, cells were stained with 7-AAD and analysed by flow cytometry. Representative example (n=2). (**D**) Previously published Vpr targets are not depleted in SLF2 KO cells. Lysates from WT Jurkat T cells, clonal SLF2 KO cells and WT Jurkat cells transduced with control or Vpr VLPs were analysed by immunoblotting 24 hpi. Representative blot (n=2). (**E**) HA-Vpr blot for primate lentiviral Vpr panel. Lysates from GFP^{+} FACS-purified primate lentiviral Vpr expressing cells were analysed by immunoblotting.

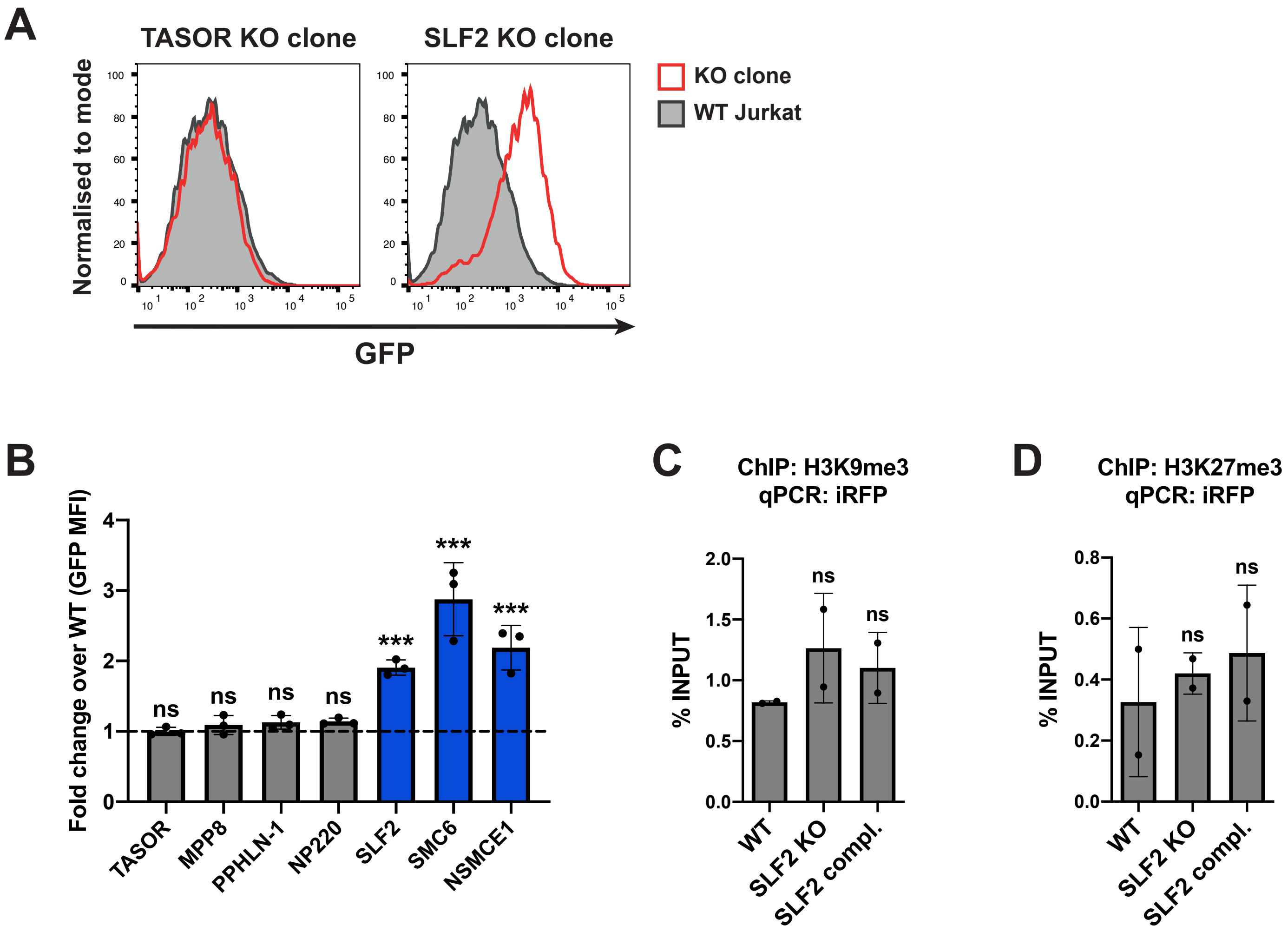

Figure S6. Unintegrated lentivirus is not restricted by the HUSH complex, and restriction does not involve H3K9me3 and H3K27me3 heterochromatin marks — Related to Figure 5.

(**A-B**) Unintegrated virus gene expression is not increased by knockout of the HUSH complex. (**A**) Unintegrated reporter virus assay in clonal TASOR and SLF2 knockout cell lines. Flow cytometry 72 hpi. Representative histograms (n=2). (**B**) Unintegrated virus reporter infection of mixed KO populations 7 days post sgRNA transduction of Cas9-Jurkat. Flow cytometry 72 hpi quantified as fold change GFP MFI over WT Jurkat. Each bar represents data for 3 independent sgRNAs with SD error bars. ns, P>0.05. ***, P<0.001. Representative experiment of n=2. (**C-D**) Classical silencing marks H3K9me3 and H3K27me3 on unintegrated virus are unaffected by SLF2 knockout. WT, clonal SLF2 knockout and SLF2 complemented SLF2 knockout cells were infected with SFFV-iRFP reporters in presence of RAL. 48 hpi, ChIP was performed using antibodies against (**C**) H3K9me3 and (**D**) H3K27me3. qPCR data from each ChIP experiment was calculated as the percentage of input DNA. Histograms summarise data from n=2 independent experiments, showing mean with standard deviation error bars. ns, P>0.05.

A

Read counts for ATAC-seq libraries

Library ID	Reads QC	Reads mapped	Duplicate reads	Reads filtered
WT Jurkat (uninfected)	156,374,826	154,908,624	16,990,686	122,393,380
No VLP (infected))	139,662,646	138,380,552	11,511,887	112,144,618
Control VLP (infected)	141,066,930	139,730,692	11,851,171	113,753,238
Vpr VLP (infected)	160,112,840	157,539,241	18,096,139	122,200,958

B

Fragment size profile

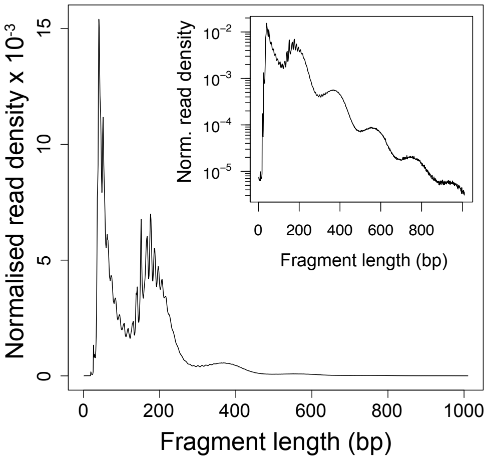

C

Read alignment for ATAC-seq libraries

	Number of mapped reads	
	**Human genome**	**Viral genome**
WT Jurkat (uninfected)	122,393,024	356
No VLP (infected)	112,124,981	19,637
Control VLP (infected)	113,731,863	21,375
Vpr VLP (infected)	122,157,863	43,095

Figure S7. ATAC-seq QC — Related to Figure 6

(**A**) Read count statistics for data filtering of ATAC-seq libraries. (**B**) Fragment size profile for ATAC-seq library (representative example) with characteristic nucleosome phasing of fragment length. (**C**) Summary of read alignment to human genome (hg38) and viral genome (1-LTR circle) for ATAC-seq libraries.
